# A prognostic risk model based on DNA methylation levels of genes and lncRNAs in lung squamous cell carcinoma

**DOI:** 10.7717/peerj.13057

**Published:** 2022-03-24

**Authors:** Weiqing Wang, Ming Xiang, Hui Liu, Xiao Chu, Zhaoyun Sun, Liang Feng

**Affiliations:** Department of Thoracic Surgery, The Fifth People’s Hospital of Shanghai, Shanghai, China

**Keywords:** Coronavirus disease 2019, Lung squamous cell carcinoma, Recurrence-free survival, Prognosis, DNA methylation

## Abstract

**Background:**

Recurrence is a risk factor for the prognosis of lung squamous carcinoma (LUSC). DNA methylation levels of RNAs are also associated with LUSC prognosis. This study aimed to construct a prognostic model with high performance in predicting LUSC prognosis using the methylation levels of lncRNAs and genes.

**Methods:**

The differentially expressed RNAs (DERs) and differentially methylated RNAs (DMRs) between the recurrent and non-recurrent LUSC tissues in The Cancer Genome Atlas (TCGA; training dataset) were identified. Weighted correlation network analysis was performed to identify co-methylation networks. Differentially methylated genes and lncRNAs with opposite expression-methylation levels were used for the screening of prognosis-associated RNAs. The prognostic model was constructed and its performance was validated in the GSE39279 dataset.

**Results:**

A total of 664 DERs and 981 DMRs (including 972 genes) in recurrent LUSC tissues were identified. Three co-methylation modules, including 226 differentially methylated genes, were significantly associated with LUSC. Among prognosis-associated RNAs, 18 DERs/DMRs with opposite methylation-expression levels were included in the methylation prognostic risk model. LUSC patients with high risk scores had a poor prognosis compared with patients who had low risk scores (TCGA: HR = 3.856, 95% CI [2.297–6.471]; GSE39279: HR = 3.040, 95% CI [1.435–6.437]). This model had a high accuracy in predicting the prognosis (AUC = 0.903 and 0.800, respectively), equivalent to the nomogram model inclusive of clinical variables.

**Conclusions:**

Referring to the methylation levels of the 16-RNAs might help to predict the survival outcomes in LUSC.

## Introduction

Lung squamous carcinoma or lung squamous cell carcinoma (LUSC) is a histologic type of lung cancer that ranks first in the rate of incidence and mortality (Bray et al., 2018). The number of newly diagnosed cases and cancer-related death of lung cancer in 2018 was approximately 2.1 million and 1.8 million, respectively (Bray et al., 2018). Non-small cell lung cancer (NSCLC) is the most predominant type of lung cancer (accounting for 80%) and LUSC composes ~30% of NSCLC (Lemjabbar-Alaoui et al., 2015).

Patients with recurrent LUSC have a very poor survival prognosis. Tumor recurrence is distinctly influenced by clinical characteristics including patients’ age, histologic type, stage, treatment response, gene expression, and epigenetic regulation (Dziedzic et al., 2016; Feng et al., 2015; Smith et al., 2010). DNA methylation of genes is critical for tumorigenesis, progression, metastasis, recurrence, and resistance to therapy (Castilho, Squarize & Almeida, 2017; Liu et al., 2017; Ma, Chen & Petersen, 2017; Sosa, Bernstein & Aguirre-Ghiso, 2017. For instance, the promoter methylation levels of secreted frizzled-related protein genes have a high accuracy in diagnosing NSCLC (Liu et al., 2017). Also, DNA methylation levels of tumor suppressor genes, including death-associated protein kinase 1 (DAPK1) and retinoic acid receptor beta (RARB), are higher in lung cancer tissues than noncancerous lung tissues (Daniunaite et al., 2020). Also, the aberrant hypermethylation levels of RARB and DAPK1 promoters are strongly associated with the invasive progression in cervical cancer (Tawe et al., 2020). Furthermore, DNA methylation-mediated regulation of long non-coding RNAs (lncRNAs) also plays crucial roles in tumors by regulating the expression of genes and microRNAs (Heilmann et al., 2017; Ma et al., 2017)

The hypomethylated promoter of the angiotensin-converting enzyme 2 (ACE2) gene, a receptor of severe acute respiratory syndrome coronavirus 2 (SARS-CoV-2), has been identified in lung adenocarcinoma tissues (Chai et al., 2020). Most of the coronavirus disease 2019 (COVID-19)-affected patients are patients who have malignancies (Calabrò et al., 2020; Luo et al., 2020). Also, lung adenocarcinoma patients are more susceptible to SARS-CoV-2 infection than LUSC patients (Kong et al., 2020). COVID-19 patients who have a history of radiation therapy for cancers appear to have a poor prognosis (Robilotti et al., 2020). COVID-19 pandemic has brought great challenges not only to lung cancer therapy (Calabrò et al., 2020; Guckenberger et al., 2020; Passaro et al., 2020) but also to the diagnosis and prognosis of lung cancer (Maringe et al., 2020; Pruis et al., 2020; Robilotti et al., 2020). These studies show a potential correlation between an increased risk of COVID-19 infection in patients with lung cancer. Therefore, the identification of more and potential biomarkers in lung cancer might provide additional information on making treatment strategies for lung cancer.

This study aimed to screen a prognostic risk score system based on DNA methylation levels of genes and lncRNAs between recurrent and non-recurrent LUSC using bioinformatics analysis. The performance of this model in predicting the prognosis of LUSC was validated using a microarray dataset. Also, a combined nomogram model to predict the prognosis in LUSC patients was constructed. This study might provide a reference for assessing the prognosis of patients with LUSC.

## Materials and Methods

### Datasets

The Cancer Genome Atlas (TCGA; https://www.cancer.gov/about-nci/organization/ccg/research/structural-genomics/tcga) LUSC RNA-seq (Illumina HiSeq 2000 RNA Sequencing; *n* = 550) and methylation profile data (Illumina Infinium Human Methylation 450 BeadChip; *n* = 415) were downloaded. After matching sample IDs, 293 samples with gene expression profiles, methylation profiles, and clinical recurrence information (with recurrence = 78; without recurrence = 215) were retained and used as the training dataset in this study.

The methylation microarray dataset GSE39279 was downloaded from the Gene Expression Omnibus (GEO) at the National Center for Biotechnology Information (NCBI; https://www.ncbi.nlm.nih.gov/geo/). It was selected using the following criteria: (1) methylation profiles from patients with LUSC; (2) inclusive of clinical recurrence information; (3) ≥150 samples. GSE39279 (GPL13534, Illumina HumanMethylation450 BeadChip) contains 444 samples, including 43 samples with recurrence information. This dataset was used as the validation dataset.

### RNA annotation and identification of RNAs differentially expressed and methylated in recurrent LUSC

The annotation of lncRNAs and mRNAs in the expression and methylation files was performed using the HUGO Gene Nomenclature Committee (HGNC; http://www.genenames.org/). Ensembl IDs were converted to official gene symbols. The differentially expressed RNAs (DER) and differentially methylated RNAs (DMR), including lncRNAs and genes, between the samples with and without recurrence were identified in the TCGA training dataset. The limma package (version 3.34.7; https://bioconductor.org/packages/release/bioc/html/limma.html) in R was used for the identification of DERs and DMRs. To maximize the intersection of DERs and DMRs, we set *p* < 0.05, false discovery rate (FDR) < 0.05, and |log_2_(Fold change, FC)| > 0.263 as the criteria for significant difference. The expression profiles of RNAs with differential expression and methylation levels were presented using the bidirectional hierarchical clustering heatmap by pheatmap (version 1.0.8; https://cran.r-project.org/web/packages/pheatmap/index.html). Besides, RNAs with both differential methylation and expression levels were identified using the Venn diagram.

### Weighted correlation network analysis (WGCNA) of genes

The WGCNA networks identify gene modules associated with disease status based on the expression/methylation profiles of genes. The WGCNA package (version 1.63; https://cran.r-project.org/web/packages/WGCNA/index.html) in R was used to analyze the co-methylation networks for RNAs in the TCGA training dataset, irrespective of the expression and methylation level. The criteria for WGCNA module identification were: min size = 100, cutHeight = 0.995, *p* < 0.05, and enrichment fold > 1. Genes included in WGCNA modules were matched with the RNAs with differentially expression and methylation levels, and the overlapping items were used for further analysis.

### Construction of methylation prognostic model

Genes and lncRNAs included in WGCNA co-methylation modules that had opposite levels of methylation and expression between the TCGA LUSC samples with and without recurrence were used as candidates for screening prognosis-associated RNAs. The univariate Cox regression analysis in the R Survival package (version 2.41-1; http://bioconductor.org/packages/survivalr/) was used for the screening of RNAs associated with LUSC prognosis. The criterion was log-rank *p* value < 0.05. The optimal methylation combination was subsequently identified using the Cox-Proportional Hazards (Cox-PH) model (L1-penalized least absolute shrinkage and selection operator, LASSO) in the R penalized package (version 0.9-50, http://bioconductor.org/packages/penalized/). The methylation prognostic model was then established and the prognostic risk score of each individual was calculated using the following algorithm: Prognostic risk score = ∑coef_DMRs_ × Methylation_DMRs_, where coef represents the coefficient (LASSO coef) of each gene identified by the Cox-PH model and Methylation notes the methylation level of the gene in each sample, respectively. For the Kaplan–Meier survival curve analysis (version 2.41-1), samples in the training and validation datasets were grouped into the high and low-risk groups according to the median prognostic risk score. Also, the receiver operating characteristic (ROC) curve analysis was performed using the R pROC (version 1.14.0; https://cran.r-project.org/web/packages/pROC/index.html) to evaluate the accuracy of using this methylation prognostic model in predicting LUSC prognosis.

### Identification of clinical variables associated with LUSC prognosis

This study also identified clinical variables associated with the LUSC prognosis in the TCGA training dataset. The associations of prognostic risk score and clinical variables (including patient age, gender, pathologic TNM classification, tumor stage, and radiation/targeted therapy) with LUSC prognosis were assessed using the univariate and multivariate Cox regression analysis in the R Survival package (version 2.41-1). Independent variables that were significantly associated with LUSC prognosis were selected using the threshold of log-rank *p* value < 0.05.

### Prognostic nomogram and index for survival

Prognostic nomogram and index are widely applied for estimated survival probability among patients with cancers and other conditions (Barnholtz-Sloan et al., 2012; Gold et al., 2009). Nomogram was established using the R “RMS” package (Version 5.1-2; https://cran.r-project.org/web/packages/rms/index.html). The score of each variable was ascribed according to its weight in the nomogram, and the individualized 3- and 5-year survival probabilities were then predicted according to total points.

### Functional annotation for lncRNAs in the methylation prognostic model

Importantly, the functional annotations of differentially expressed lncRNAs including in the methylation prognostic model were identified to investigate the biological themes associated with lncRNAs. The correlation between expression profiles of differentially expressed genes and lncRNAs in recurrent LUSC samples in the TCGA training dataset were calculated using the Pearson correlation coefficient (*r*). LncRNA-gene pairs with confident coexpression levels (*r* > 0.4 or *r* < –0.4) were retained and the lncRNA-mRNA regulatory network was constructed using the Cytoscape software (version 3.8.0; http://www.cytoscape.org/). Using the expression profiles of the lncRNA-associated genes in the TCGA training samples, Kyoto Encyclopedia of Genes and Genomes (KEGG) pathways associated with lncRNAs were identified using the Gene Set Enrichment Analysis (GSEA) software (version 4.0.2; http://software.broadinstitute.org/gsea/index.jsp). The threshold for significant enrichment was a normal *p* value < 0.05.

## Results

### Identification of DERs and DMRs

**Figure 1:**
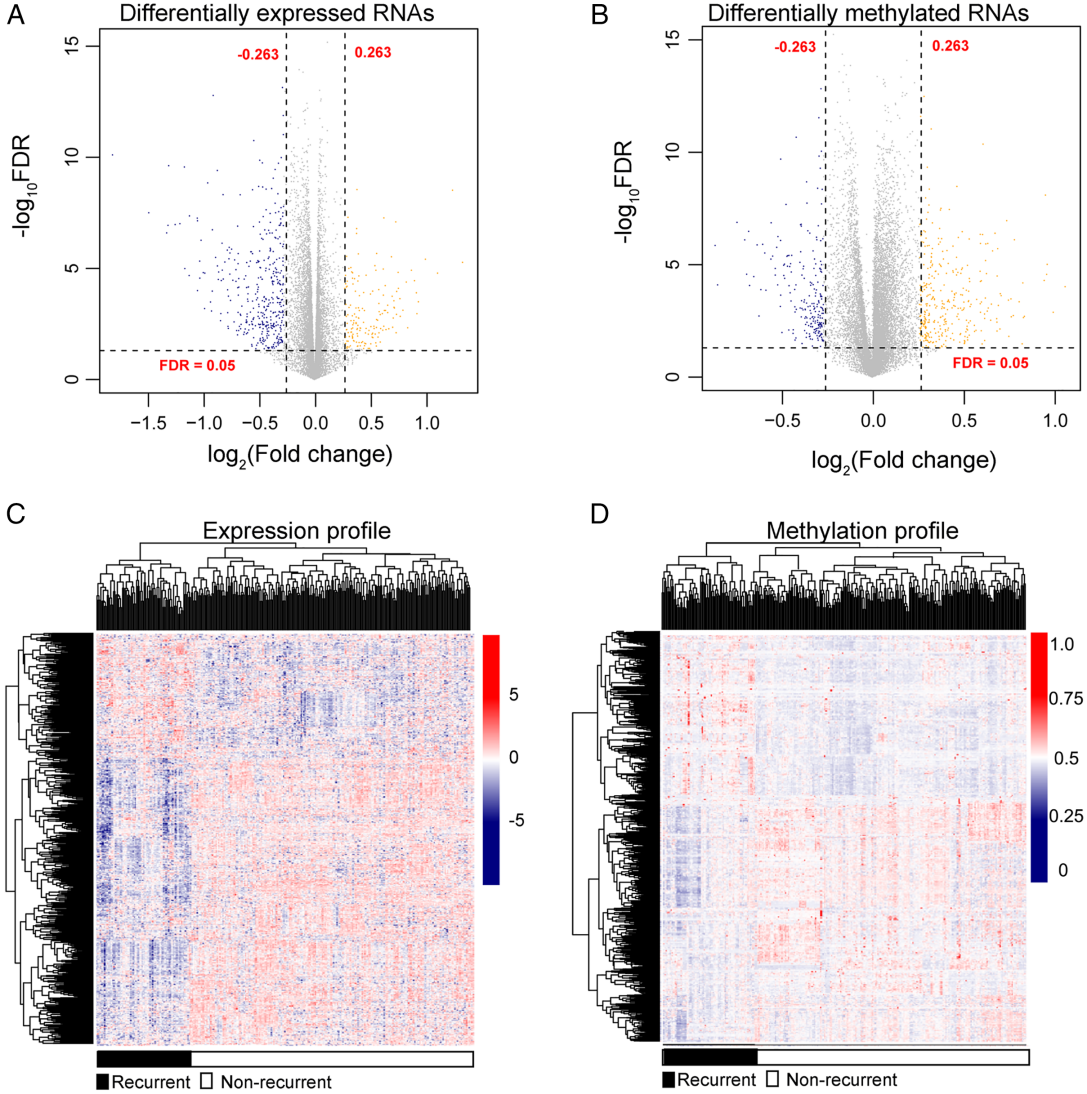
The expression and methylation levels of lncRNAs and genes in lung squamous carcinoma samples. (A & B) The Volcano plots indicating the differentially expressed and methylated RNAs between recurrent and non-recurrent tumors. (C & D) The bidirectional hierarchical clustering heatmap indicating the expression (log_2_[FPKM + 1]) and methylation profiles (beta value) of RNAs. FDR, false discovery rate.

**Figure 2:**
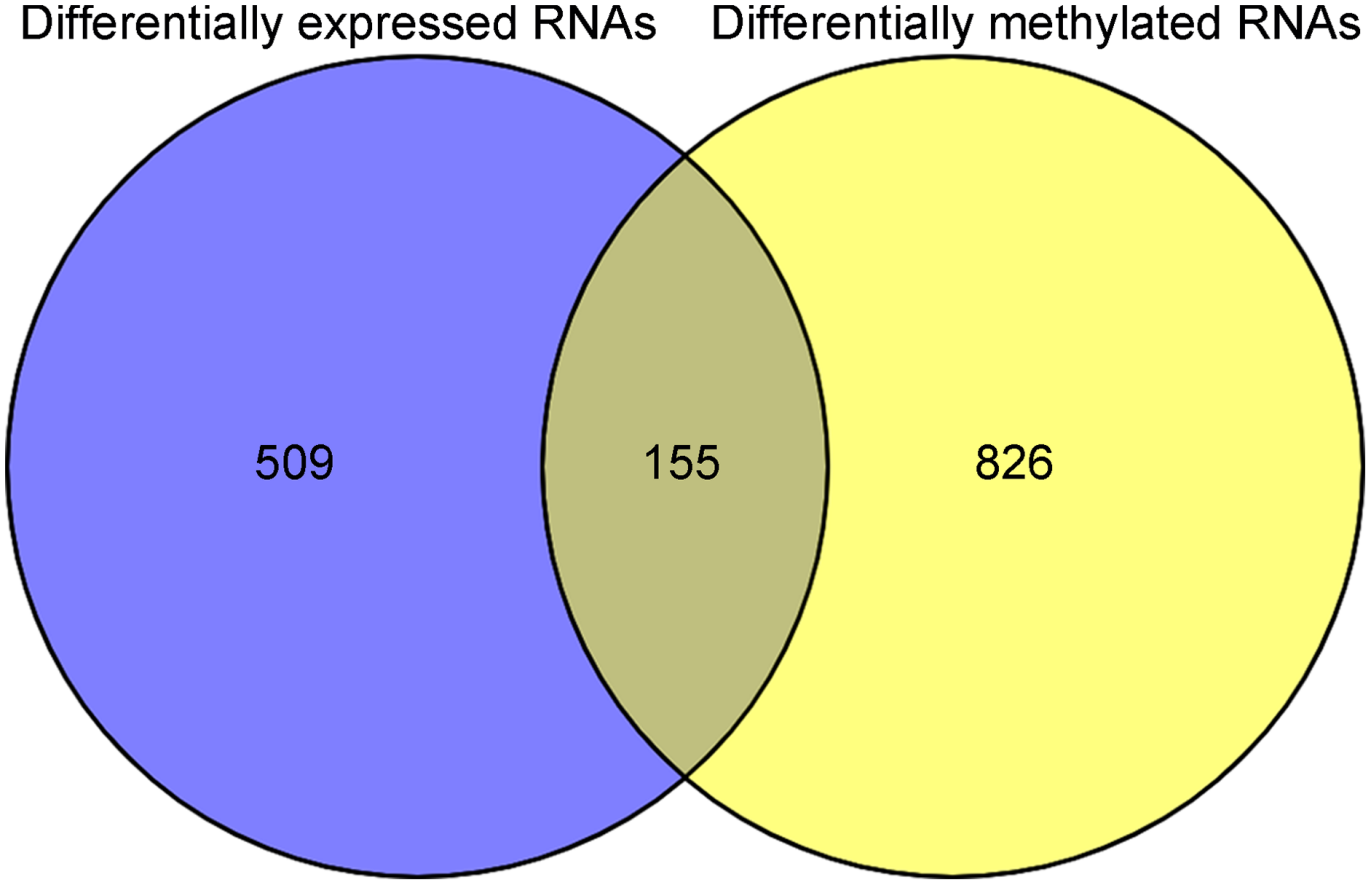
The Venn diagram indicating the RNAs with differentially expression and methylation levels in recurrent lung squamous carcinoma samples.

After annotation in the HGNC database, a total of 54 lncRNAs and 12,932 genes with available expression profiles were obtained in the TCGA dataset, including 664 DERs and 981 DMRs (including 972 genes and nine lncRNAs) between the LUSC samples with and without recurrence (Figs. 1A and 1B). The expression and methylation profiles of the DERs and DMRs in the LUSC samples are shown in Figs. 1C and 1D, respectively. Venn diagram showed that 155 DERs were differentially methylated in recurrent LUSC samples compared with samples without tumor recurrence (Fig. 2; Table S1).

### WGCNA for DERs with differentially methylation levels in LUSC samples

**Figure 3:**
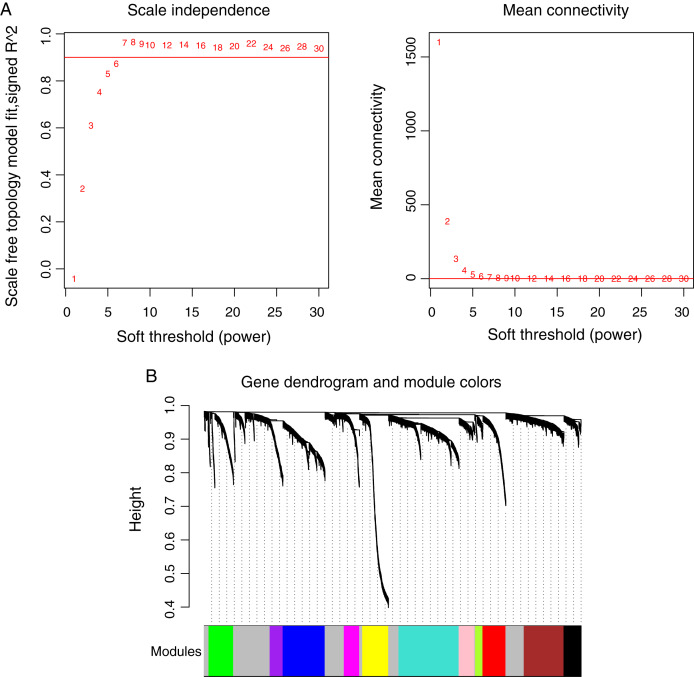
Weighted correlation network analysis (WGCNA) for RNAs with differentially methylation and expression levels. (A) Plots showing the selection of the soft-thresholding power (*n* = 7). (B) The gene dendrogram indicating the identification of WGCNA modules.

**Figure 4:**
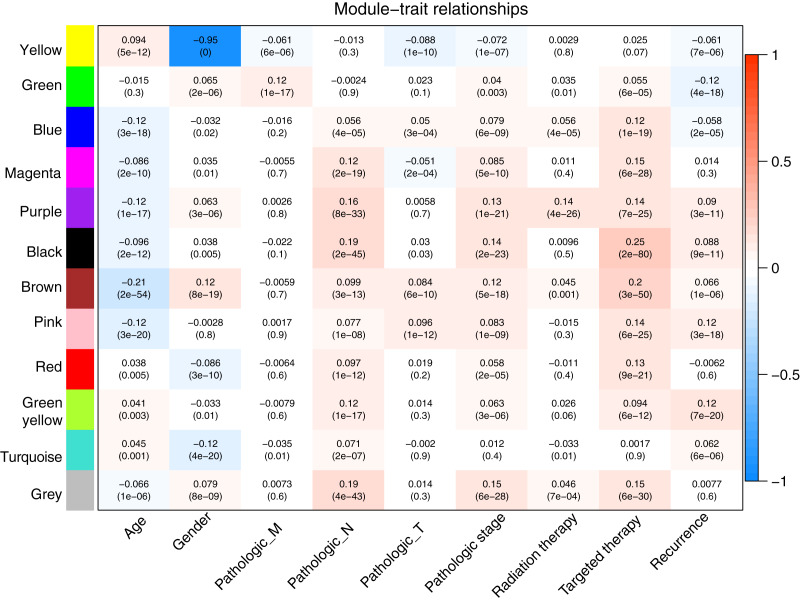
Heatmap showing the correlation of WGCNA modules with clinical variables. Red and blue notes positive (maximum value = 1) and negative (minimum value = –1) correlation, respectively. WGCNA, weighted correlation network analysis.

Prior to the WGCNA module analysis, the scale-free topology prerequisite soft-thresholding power was identified. It was 7 when the scale-free topology model fit *R*^2^ = 0.9 for the first time (Fig. 3A). The mean gene connectivity = 1 when soft-thresholding power = 7 (Fig. 3B), conforming to the scale-free network property. Based on the methylation profiles of all RNAs, 12 WGCNA modules (including 105-1327 RNAs) were subsequently identified in the TCGA training dataset according to the criteria we set before (soft threshold power = 7, min size = 100, and cutHeight = 0.995; Fig. 3C; Table 1). The module-trait relationship heatmap showing the correlations of modules with clinical variables is shown in Fig. 4. Also, the numbers of differentially methylated genes and fold enrichment value of each module are shown in Table 1. Three WGCNA co-methylation modules (green, pink, and turquoise), including 226 differentially methylated genes, had enrichment folds of >1 and an enrichment *p* value of <0.05.

**Table 1:** Weighted correlation network analysis (WGCNA) module information.

Module	Size	DMGs	Enrichment information	
			Enrichment fold (95% CI)	P_hyper_
Black	245	10	0.387 (0.182–0.729)	1.30E−03
Blue	601	58	0.914 (0.677–1.217)	5.75E−01
Brown	570	47	0.781 (0.561–1.068)	1.27E−01
**Green**	**352**	**99**	**2.665 (2.076**–**3.398)**	**8.41E−14**
Greenyellow	105	5	0.451 (0.143–1.093)	9.72E−02
Grey	1,327	135	0.964 (0.786–1.176)	7.65E−01
Magenta	220	21	0.905 (0.544–1.431)	7.38E−01
**Pink**	**224**	**36**	**1.523 (1.029**–**2.198)**	**3.16E−02**
Purple	186	4	0.204 (0.0547–0.533)	1.08E−04
Red	329	33	0.951 (0.637–1.378)	8.54E−01
**Turquoise**	**861**	**91**	**1.201 (1.046**–**2.267)**	**4.96E−02**
Yellow	372	28	0.713 (0.463–1.060)	9.28E−02

**Notes:**

DMG, differentially methylated genes; CI, confident interval; P_hyper,_ *p* value by hypergeometric algorithm. Bold text notes significant modules and parameters.

### Identification of LUSC prognosis-associated RNAs

After matching the above 226 WGCNA module genes with the 155 RNAs in Fig. 2, 41 common genes were identified (Table S3). Among them, 28 genes had opposite methylation-expression levels in the TCGA LUSC samples (Table S3). Besides, six of the nine lncRNAs listed in Table S1 had opposite methylation-expression levels. Among the 34 candidate DERs (including 28 genes and six lncRNAs), 26 DERs (including four lncRNAs and 22 genes) were identified as prognosis-associated RNAs by univariate Cox regression analysis and methylation profiles (Table S4).

### Construction of the methylation prognostic model

An optimized methylation prognostic combination was identified using the Cox-PH model based on the 26 prognosis-associated DMRs. Eighteen DMRs, including two lncRNAs and 16 genes, were included in this methylation prognostic combination (Table 2). The associations of seven RNAs with LUSC prognosis by the multivariate Cox PH model (Table 2), including *DIRC3*, *RMST*, *ABCA12*, *ADH7*, *DGKA*, *NPHP3*, and *WFDC10B*, were inconsistent with those analyzed by the univariate Cox regression analysis in R package (Table S4). Accordingly, the methylation prognostic model was constructed and the prognostic risk score of each sample was calculated as: (–0.0103) × Methylation_*DIRC3*_ + (–0.0242) × Methylation_*RMST*_ + 0.0866 × Methylation_*LTF*_ + 0.0438 × Methylation_*SGCG*_ + (–0.0476) × Methylation_*ABCA12*_ + (–0.0171) × Methylation_*ADH7*_ + 0.0846 × Methylation_*BNIPL*_ + (–0.0273) × Methylation_*DGKA*_ + 0.1114 × Methylation_*FAM181B*_ + 0.0376 × Methylation_*GNRH2*_ + 0.0575 × Methylation_*HORMAD2*_ + (–0.7505) × Methylation_*LIMCH1*_ + (–0.0164) × Methylation_*NPHP3*_ + 0.0823 × Methylation_*RTP1*_ + 0.1422 × Methylation_*ST6GALNAC1*_ + 0.0667 × Methylation_*THNSL2*_ + 0.0546 × Methylation_*TRIM7*_ + (–0.0604) × Methylation_*WFDC10B*_. Almost all these genes, except *LIMCH1* (expression logFC = 0.27, methylation logFC = –0.39), were downregulated and hypermethylated in recurrent LUSC samples compared with non-recurrent LUSC samples (Tables S1 and S2).

**Table 2:** RNAs used for the construction of the prognostic methylation model in this study.

Symbol	Type	Multivariate Cox regression analysis			LASSO coef
		HR	95% CI	*p* value	
*DIRC3*	lncRNA	0.932	0.779–0.996	3.880E−02	–0.0103
*RMST*		0.961	0.830–0.985	1.140E−02	–0.0242
*LTF*	mRNA	1.276	1.101–3.946	2.080E−02	0.0866
*SGCG*		1.198	1.054–5.930	4.170E−02	0.0438
*ABCA12*		0.925	0.769–0.986	4.120E−02	–0.0476
*ADH7*		0.965	0.797–0.976	4.140E-02	–0.0171
*BNIPL*		1.349	1.112–5.9131	2.630E−02	0.0846
*DGKA*		0.864	0.684–0.937	3.220E−02	–0.0273
*FAM181B*		1.585	1.199–3.864	2.550E−02	0.1114
*GNRH2*		1.194	1.029–3.888	2.690E−02	0.0376
*HORMAD2*		1.380	1.114–4.899	4.130E−02	0.0575
*LIMCH1*		0.401	0.257–0.624	5.280E−06	–0.7505
*NPHP3*		0.953	0.800–0.986	4.420E−02	–0.0164
*RTP1*		1.363	1.118–5.918	3.070E−02	0.0823
*ST6GALNAC1*		1.491	1.190–3.950	1.100E−02	0.1422
*THNSL2*		1.224	1.080–4.954	1.980E−02	0.0667
*TRIM7*		1.289	1.097–4.934	2.940E−02	0.0546
*WFDC10B*		0.895	0.728–0.912	3.640E−02	–0.0604

**Note:**

LASSO, L1-penalized least absolute shrinkage and selection operator; HR, hazard ratio; CI, confident interval; coef, coefficient.

### Accuracy of the methylation prognostic model in predicting prognosis in LUSC

**Figure 5:**
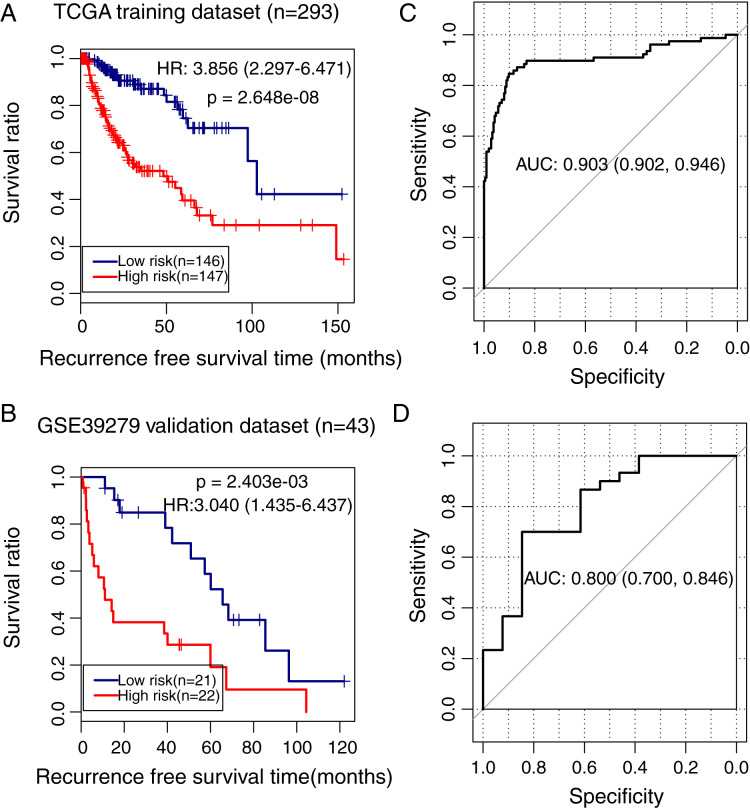
Analysis of the methylation prognostic model in lung squamous cell carcinoma. (A & B) The Kaplan–Meier curves showing the differences in recurrence-free survival ratios between patients with high- and low-risk scores in the training (TCGA) and validation (GSE39279) dataset, respectively. Prognostic risk scores were calculated based on the methylation prognostic model. (C & D) The receiver operating characteristic (ROC) curve analysis for this model in the training and validation cohort, respectively. AUC, area under the ROC curve.

To validate the predictive power of the methylation prognostic model in LUSC, samples in the TCGA training dataset (*n* = 293) and the GSE39279 validation dataset (*n* = 43) were separately grouped into the high and low-risk groups according to the median risk score of each sample. Kaplan–Meier survival curve analysis showed that there was a significant difference in the recurrence-free survival ratio between patients with high and low-risk scores in the TCGA training dataset (HR = 3.856, 95% CI [2.297–6.471], *p* = 2.648e − 08; Fig. 5A) and the GSE39279 validation dataset (HR = 3.040, 95% CI [1.435–6.437], *p* = 2.403e − 03; Fig. 5B). The ROC curve analysis showed that this methylation prognostic model had a high accuracy in predicting LUSC prognosis in the training dataset (area under the ROC curve, AUC = 0.903, 95% CI [0.902–0.946]; Fig. 5C) and in the validation dataset (AUC = 0.800, 95% CI [0.700–0.846]; Fig. 5D).

### Clinical variables associated with LUSC prognosis

**Figure 6:**
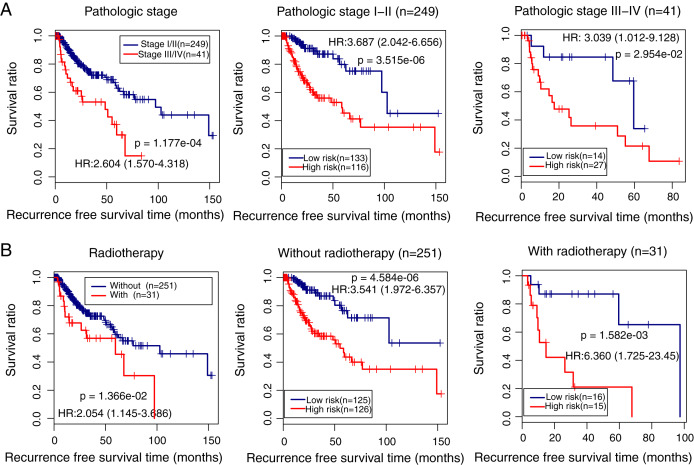
Subgroup analysis showing the performance of the methylation prognostic model in predicting prognosis in lung squamous carcinoma. (A & B) The differences in the survival ratio between patients with different clinical variables (left) and between patients with high- and low-risk scores (middle and right). HR, hazards ratio.

We also identified the clinical variables associated with the prognosis of LUSC in the TCGA cohort. Univariate and multivariate Cox regression analysis confirmed that pathologic stage (HR = 1.443, 95% CI [1.297–2.854], *p* = 2.92e − 02), radiation therapy (HR = 1.963, 95% CI [1.022–3.771], *p* = 4.27e − 02), and prognostic risk score (HR = 3.874, 95% CI [2.256–6.653], *p* = 9.12e − 07) were associated with LUSC prognosis (Table 3). Kaplan–Meier curve analysis showed that patients with advanced stages and with radiotherapy had a lower recurrence-free survival ratio compared with controls (pathologic stage: HR = 2.604, 95% CI [1.570–4.318], *p* = 1.177e − 04; Fig. 6A, left; radiotherapy: HR = 2.054, 95% CI [1.145–3.686], *p* = 1.36e − 02; Fig. 6B, left). Subgroup survival analysis showed that patients with high prognostic risk scores (high-risk group) had a lower survival ratio compared with patients who had low scores (low-risk group), irrespective of pathologic stage and radiotherapy (Figs. 6A and 6B, middle and right).

**Table 3:** Cox regression analysis for clinical variables in The Cancer Genome Atlas (TCGA) lung squamous cell carcinoma cohort.

Clinical characteristics	TCGA ( *n* = 293)	Univariate		Multivariate	
		HR (95% CI)	*p* value	HR (95% CI)	*p* value
Age (years, mean ± SD)	67.72 ± 8.63	0.988 (0.962–1.014)	3.51E−01		
Gender (Male/Female)	211/82	1.329 (0.783–2.257)	2.90E−01		
Pathologic M (0/1/–)	227/2/64	0.303 (0.151–1.536)	6.07E−01		
Pathologic N (0/1/2/–)	193/76/20/4	1.562 (1.142–2.138)	4.72E−03	1.011 (0.519–1.854)	9.53E−01
Pathologic T (1/2/3/4)	79/159/48/7	1.639 (1.209–2.220)	1.43E−03	1.223 (0.749–1.998)	4.21E−01
Pathologic stage (I/II/III/IV/–)	139/110/39/2/3	1.768 (1.326–2.356)	7.57E−05	1.443 (1.297–2.854)	2.92E−02
Radiation therapy (Yes/No/–)	31/251/11	2.054 (1.145–3.686)	1.37E−02	1.963 (1.022–3.771)	4.27E−02
Targeted therapy (Yes/No/–)	94/188/11	1.238 (0.774–1.981)	3.72E−01		
PS status (High/Low)	146/147	3.856 (2.297–6.471)	2.65E−08	3.874 (2.256–6.653)	9.12E−07
Recurrence (Yes/No)	78/215				
RFS time (months, mean ± SD)	28.28 ± 27.45				

**Note:**

CI, confident interval; HR, hazard ratio; SD, standard deviation; RFS, recurrence-free survival; PS, prognostic risk score.

### Prognostic nomogram

**Figure 7:**
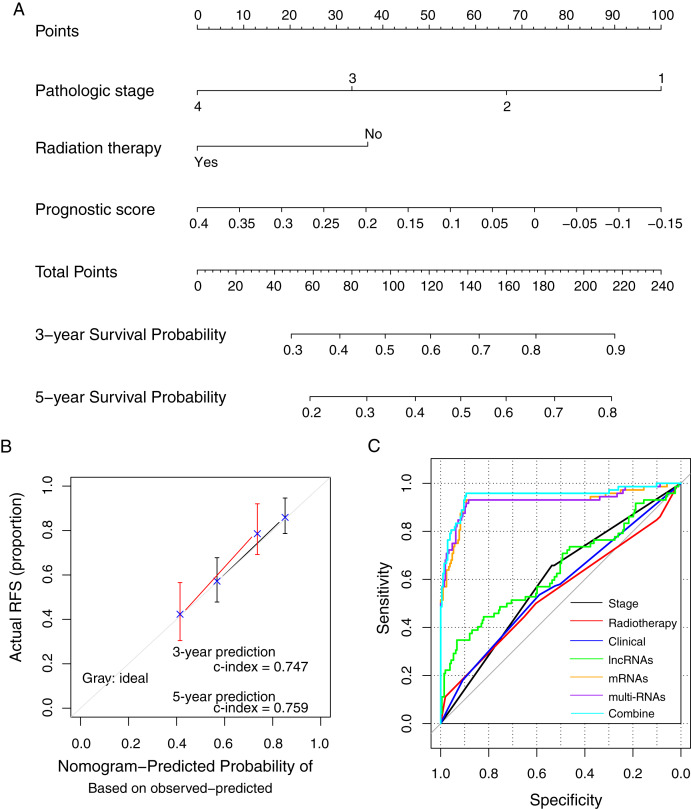
The prognostic nomogram and index for recurrence-free survival. (A) The weight of clinical factors and prognostic risk score in predicting the prognosis of lung squamous carcinoma. (B) The c-index analysis for the predicted survival probability based on the nomogram model. (C) The receiver operating characteristic (ROC) curve analysis for all models. RFS, recurrent-free survival.

The prognostic nomogram was constructed using the methylation prognostic model, pathologic stage (1–4), and radiation therapy (yes/no; Fig. 7A). The predicted 3- and 5-year survival probabilities based on the nomogram had high compliances with the actual situations (Fig. 7B; c-index = 0.747 and 0.759 for 3-year and 5-year prediction, respectively). For an example case with stage III tumor (points = 33.3), radiation therapy (points = 0), and a risk score of 0.2 (points = 37.7; total points = 71), the predicted 3- and 5-year survival probability of the case based on the nomogram model was about 38.7% and 23.9%, respectively. ROC curve analysis showed that the combination of the clinical variables with the methylation prognostic model had the highest AUC value (AUC = 0.948, 95% CI [0.894–0.958]) and c-index (0.755; *p* = 2.22e − 16; Fig. 7C; Table 4), followed by the methylation prognostic model alone (AUC = 0.927, 95% CI [0.884–0.931], c-index = 0.739, *p* = 6.66e − 15).

**Table 4:** The receiver operating characteristic (ROC) curve analysis for the clinical variables and prognostic methylation model.

Type	AUC	C-index	*p* value
Stage	0.596 (0.537, 0.658)	0.653	4.64E−06
Radiotherapy	0.548 (0.604, 0.500)	0.550	6.41E−02
Clinical	0.598 (0.568, 0.536)	0.656	8.38E−06
lncRNAs	0.640 (0.732, 0.547)	0.542	2.77E−01
mRNAs	0.924 (0.894, 0.931)	0.732	7.04E−14
multi-RNAs	0.927 (0.884, 0.931)	0.739	6.66E−15
Combine	0.948 (0.894, 0.958)	0.755	2.22E−16

**Note:**

AUC, area under the ROC curve.

### Pathways associated with the two lncRNAs in the methylation prognostic model

**Figure 8:**
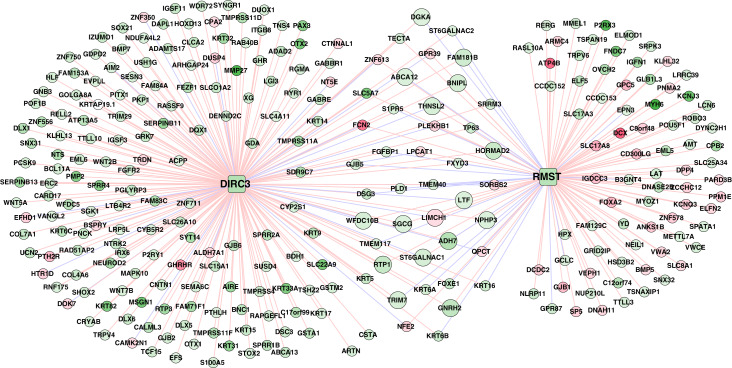
The lncRNA–mRNA network of *DIRC3* and *RMST*. This network was constructed using the differentially expressed genes that had confident correlations (*r* > 0.4 or *r* < –0.4) with the two lncRNAs in lung squamous carcinoma samples. Green and red color indicate downregulation and upregulation, respectively. Red and blue lines note positive (*r* > 0.4) and negative (*r* < –0.4) correlations, respectively.

A total of 320 lncRNA-mRNA pairs related to the two lncRNAs (*DIRC3* and *RMST*, downregulation and hypermethylation) in the methylation prognostic model were extracted (*r* > 0.4 or *r* < –0.4; Table S5). Accordingly, the lncRNA-mRNA network included 320 interactions and 281 nodes (two lncRNAs and 279 differentially expressed genes; Fig. 8).

Downregulated genes with hypermethylation levels, including *LTF*, *ADH7*, *ST6GALNAC1*, *THNSL2*, and *WFDC10B*, had positive correlations with *RMST*, genes including *ABCA12*, *DGKA*, *FAM181B*, and *TRIM7* were positively correlated with *DIRC3*, *LIMCH1* had negative correlation with both of *RMST* and *DIRC3*, and the other genes including *SGCG*, *BNIPL*, *GNRH2*, *HORMAD2*, *NPHP3*, and *RTP1* had positive correlations with both of *RMST* and *DIRC3* (Table S5). Based on the expression profiles of the 279 genes in the TCGA LUSC cohort, we identified that *DIRC3* and *RMST* were associated with five and four KEGG pathways, respectively (Table 5). *DIRC3* and *RMST* both were associated with the “Pathways in cancer” and “Endocytosis”.

**Table 5:** Pathways associated with lncRNA DIRC3 and RMST.

Name	Size	ES	NES	NOM *p*-value	Gene
*lncRNA DIRC3*					
Metabolism of xenobiotics by cytochrome P450	4	0.82	1.65	8.14E−03	*CYP2S1, GSTM2, GSTA1, ADH7*
Drug metabolism cytochrome P450	3	0.82	1.51	2.75E−02	*GSTM2, GSTA1, ADH7*
Endocytosis	3	0.74	1.50	3.37E−02	*GRK7, PLD1, FGFR2*
MAPK signaling pathway	2	0.64	1.31	4.14E−02	*NTRK2, FGFR2*
Pathways in cancer	4	0.59	1.44	4.82E−02	*WNT2B, WNT5A, COL4A6, FGFR2*
*lncRNA RMST*					
Pathways in cancer	5	–0.62	–1.64	5.03E−03	*WNT7B, WNT5A, COL4A6, PLD1, FGFR2*
Neurotrophin signaling pathway	2	–0.76	–1.37	1.20E−02	*NTRK2, CALML3*
Endocytosis	4	–0.67	–1.37	1.55E−02	*EPN3, GRK7, PLD1, FGFR2*
Wnt signaling pathway	2	–0.52	–1.18	2.88E−02	*WNT7B, WNT5A*

**Note:**

ES, enrichment score; NES, normalized enrichment score; Nom *p*-value, normal *p* value.

## Discussion

Our study constructed a methylation prognostic model consisting of 18 DERs, including two lncRNAs (*DIRC3* and *RMST*) and 16 genes (*LTF*, *ADH7*, *ST6GALNAC1*, *LIMCH1*, *THNSL2*, *WFDC10B*, *SGCG*, *BNIPL*, *GNRH2*, *HORMAD2*, *ABCA12*, *DGKA*, *FAM181B*, *TRIM7*, *NPHP3*, and *RTP1*), based on the recurrent status in LUSC patients. Among the 18 RNAs, 17 DERs were downregulated with hypermethylation levels in recurrent LUSC tissues compared with non-recurrent tumors. The 18-RNA methylation prognostic model and the nomogram model consisting of the 18-RNA methylation signature and clinical variables both had high performance in predicting LUSC prognosis.

The inconsistency that seven RNAs, including *DIRC3*, *RMST*, *ABCA12*, *ADH7*, *DGKA*, *NPHP3*, and *WFDC10B*, by the multivariate Cox PH model was inconsistent with the univariate Cox regression analysis in R package was identified in our study. The methylations of seven RNAs were associated with poor prognosis in LUSC patients by the univariate Cox regression analysis, but the Multivariate Cox PH model showed that they were associated with good prognosis in LUSC. However, further analyses showed that there were confident correlations among the two lncRNAs *RMST* and *DIRC3* and the other 16 genes, which could be the cause of the above inconsistency. Also, the lncRNA-mRNA network showed that the genes might be regulated by the two lncRNAs. These results showed that the two lncRNAs *RMST* and *DIRC3* might be confounding factors for the other genes.

In the methylation prognostic model, LIM and calponin-homology domains 1 (*LIMCH1*) is the only gene downregulated and positively correlated with the LUSC prognosis by univariate Cox regression analysis (HR = 7.96E–03). *LIMCH1* regulates the activity of an important motor protein monmuscle myosin II and cell migration and growth in lung cancer cells (Lin et al., 2017; Zhang, Zhang & Xu, 2019). It partially restored the invasive phenotype of cancer cells (Bersini et al., 2020). However, Halle et al. (2021) showed that high *LIMCH1* expression was significantly associated with poor survival of cervical cancer (*p* = 0.004, HR = 3.17). Also, our study showed that high *LIMCH1* methylation level contributed to a low risk score and was significantly associated with a good prognosis of LUSC. These results showed that the contribution of expression and methylation levels of *LIMCH1* and other RNAs in the methylation prognostic model to LUSC prognosis should be validated in more cohorts.

The HORMA Domain Containing 2 (*HORMAD2*) gene is a host gene of HIV proviruses and it has one provirus integration, which is essential for synapsis surveillance during host cell meiosis (Fennessey et al., 2017; Symons, Cameron & Lewin, 2018). Also, this increases the difficulty of virus removal and provides an implication for virus management (Symons, Cameron & Lewin, 2018). The tripartite motif-containing 7 (*TRIM7*) gene is a tumor suppressor gene that encodes glycogenin-interacting proteins and an E3 ubiquitin ligase, which suppresses the progression of hepatocellular carcinoma (Jin et al., 2020; Zhu et al., 2020). E3 ubiquitin ligases are involved in various cellular functions, including innate immunity and inflammation (Giraldo et al., 2020a). Loss of N6-Methyladenosine modification of *TRIM7* promoter was observed in osteosarcoma tissues (Zhou et al., 2020). Evidence shows that E3 ubiquitin ligase positively regulates toll-like receptor 4 (TLR4)-mediated immune response in macrophages (Lu et al., 2019). Of special interest are recent reports showing their roles in virus replication (Giraldo et al., 2020a; Stukalov et al., 2020).

TRIM7 can either promote virus pathogenesis or protect against infection, depending on the context of virus infection (Giraldo et al., 2020a; Orchard et al., 2019). It promotes herpes virus and Zika virions infection (Giraldo et al., 2020b) but may play an antiviral role against norovirus (Orchard et al., 2019). TRIM7-mediated ubiquitination of the envelope protein of Zika virions promotes the release of virus from infected cells and binding of envelope protein to host cellular receptors, and enhances viral entry and replication (Orchard et al., 2019). A recent study by Stukalov et al. (2020) highlighted that the TRIM7 protein binding to the SARS-CoV-2 M phosphorylation site to drive ubiquitination. However, TRIM7’s effect on SARS-CoV-2 replication is not clear now. Our present study found that the DNA methylation levels of *HORMAD2* and *TRIM7* promoter were upregulated in recurrent LUSC tissues compared with non-recurrent tissues, and *HORMAD2* and *TRIM7* expression were downregulated in recurrent LUSC tissues. The deregulation and methylation levels of *HORMAD2* and *TRIM7* in recurrent LUSC tissues might provide references for exploring the potential association of LUSC prognosis with virus infection.

LncRNAs rhabdomyosarcoma 2 associated transcript (*RMST*) and disrupted in renal carcinoma 3 (*DIRC3*) both, especially the former, are known as tumor suppressors (Coe et al., 2019; Kong, Liu & Kong, 2018; Liu et al., 2019; Wang et al., 2018). The depletion and loss of function mutation of the two lncRNAs eliminate the threat of malignant transformation, promote tumor cell proliferation, migration, and invasion (Coe et al., 2019; Liu et al., 2019; Wang et al., 2018). *RMST* could enhance tumor cell apoptosis, block the G0/G1 phase and cell proliferation, and restrain cell invasion and migration in triple-negative breast cancer (Wang et al., 2018). *RMST* (Peng et al., 2020) positively regulates the DNA methyltransferase 3B (*DNMT3B*) and negatively regulates *DNMT3* expression (Peng et al., 2020). *RMST* knockout suppresses *DNMT3* by promoting the binding of HuR protein to DNMT3B and enhancing DNMT3B stability (Peng et al., 2020). What’s more, the deletion of *Dnmt3a* in mouse promotes lung tumor progression (Gao et al., 2011), suggesting the critical roles of *RMST* in regulating tumor progression and development.

Among the downregulated and hypermethylated genes associated with both *RMST* and *DIRC3* (including *BNIPL*, *HORMAD2*, and *NPHP3*), *HORMAD2* is the only gene related to lung cancer (Liu et al., 2012). Liu et al. (2012) showed that *HORMAD2* mRNA expression was rarely expressed in the lung and HORMAD2 protein was detected more frequently in early-stage lung adenocarcinoma compared with advanced cancers. The nephronophthisis 3 (*NPHP3*) gene is associated with nephronophthisis and is necessary for the formation of primary cilia formation (Abdullah et al., 2017; Lee, Kim & Moon, 2019). BNIP-2-like (BNIPL) is an apoptosis-associated protein that interacts with cell proliferation-related proteins including BCL-2 and Cdc42GAP (Qin et al., 2003). However, our present study showed that these genes and lncRNAs were downregulated in recurrent LUSC specimens, along with increased methylation levels. Besides, the inclusion of them in the prognostic model had high performance in predicting the prognosis in LUSC patients, suggesting the novel and crucial roles of these genes in LUSC progression.

## Conclusions

In summary, our study identified a prognostic model based on the methylation levels of lncRNAs and genes in LUSC patients with and without recurrence. This methylation had high performance in predicting the prognosis as well as the 3-year and 5-year survival probabilities in LUSC patients. Of special interest is that most of these RNAs had not been reported in lung cancers and two genes (*HORMAD2* and *TRIM7*) might associate with virus infection. Referring to the methylation levels of these RNAs might help to predict the survival outcomes in LUSC.

## Supplemental Information

10.7717/peerj.13057/supp-1 Supplemental Information 1 List of the 155 genes with differentially expression and methylation levels in recurrent lung squamous cell carcinoma samples.

10.7717/peerj.13057/supp-2 Supplemental Information 2 The methylation level of the 18 genes in the GSE39279 dataset.

10.7717/peerj.13057/supp-3 Supplemental Information 3 List of the 41 genes with differentially expression and methylation levels in three weighted correlation network analysis co-methylation modules.

10.7717/peerj.13057/supp-4 Supplemental Information 4 RNAs associated with prognosis in lung squamous cell carcinoma by univariate Cox regression analysis and methylation level.

10.7717/peerj.13057/supp-5 Supplemental Information 5 The lncRNA-mRNA pairs for DIRC3 and RMST with confident correlations (Pearson correlation coefficient, r > 0.4 or < –0.4).

## Additional Information and Declarations

### Data Availability

The following information was supplied regarding data availability: The original LUSC RNA-seq (Illumina HiSeq 2000 RNA Sequencing) and methylation profile data (Illumina Infinium Human Methylation 450 BeadChip) are available at The Cancer Genome Atlas (TCGA LUSC; https://www.cancer.gov/about-nci/organization/ccg/research/structural-genomics/tcga) and the original microarray data is available at the Gene Expression Omnibus: GSE39279.
