# Supplementary material for: A prognostic risk model based on DNA methylation levels of genes and lncRNAs in lung squamous cell carcinoma
Source: PeerJ. 2022 Mar 24;10:e13057. doi: 10.7717/peerj.13057 (PMC8958968; doi:10.7717/peerj.13057)
Supplement: Supplemental Information 1 [file peerj-10-13057-s001.docx]

Table S1. List of the 155 genes with differentially expression and methylation levels in recurrent lung squamous cell carcinoma samples.

|  |  | **Methylation level** | | | **Expression level** | | | |
| --- | --- | --- | --- | --- | --- | --- | --- | --- |
| **Symbol** | **Type** | **logFC** | **FDR** | **P value** | | **logFC** | **FDR** | **P value** |
| *DIO3OS* | lncRNA | 0.32 | 4.73E-03 | 5.84E-05 | | -0.28 | 1.67E-02 | 4.11E-05 |
| *DIRC3* | lncRNA | 0.55 | 8.62E-04 | 1.06E-05 | | -0.58 | 4.29E-02 | 1.06E-04 |
| *HAR1B* | lncRNA | 0.35 | 4.92E-02 | 6.07E-04 | | 0.36 | 2.15E-02 | 5.29E-05 |
| *HCG11* | lncRNA | 0.48 | 6.73E-03 | 8.31E-05 | | 0.49 | 3.28E-02 | 8.10E-05 |
| *HCG9* | lncRNA | -0.29 | 2.64E-02 | 3.26E-04 | | -0.38 | 2.35E-02 | 5.80E-05 |
| *RMST* | lncRNA | 0.37 | 5.40E-03 | 6.67E-05 | | -0.56 | 2.22E-02 | 5.47E-05 |
| *TCL6* | lncRNA | 0.29 | 6.63E-03 | 8.19E-05 | | -0.42 | 1.65E-02 | 4.06E-05 |
| *TTTY10* | lncRNA | 0.36 | 2.32E-02 | 2.87E-04 | | -0.32 | 3.82E-02 | 9.43E-05 |
| *XIST* | lncRNA | 0.30 | 1.83E-03 | 2.26E-05 | | -0.77 | 8.20E-03 | 2.03E-05 |
| *ABCA12* | mRNA | 0.35 | 2.36E-02 | 2.92E-05 | | -0.29 | 5.20E-03 | 1.29E-05 |
| *ACER1* | mRNA | 0.27 | 1.58E-02 | 1.95E-05 | | 0.30 | 3.15E-02 | 7.76E-05 |
| *ADH7* | mRNA | 0.30 | 3.81E-02 | 4.71E-05 | | -0.57 | 2.18E-02 | 2.70E-05 |
| *AIM2* | mRNA | 0.31 | 1.92E-02 | 2.38E-05 | | -0.28 | 4.33E-03 | 1.07E-05 |
| *ALDH7A1* | mRNA | -0.55 | 9.53E-03 | 1.18E-05 | | 0.34 | 3.59E-03 | 4.43E-06 |
| *AMPD1* | mRNA | 0.29 | 1.53E-02 | 1.89E-05 | | -0.48 | 4.55E-03 | 1.12E-05 |
| *ANKS1B* | mRNA | 0.63 | 3.96E-02 | 4.89E-05 | | 0.42 | 3.04E-02 | 3.75E-05 |
| *ARL4D* | mRNA | 0.31 | 5.72E-03 | 7.06E-06 | | -0.29 | 1.16E-02 | 1.43E-05 |
| *ARMC4* | mRNA | 0.27 | 8.40E-03 | 1.04E-05 | | 0.33 | 1.46E-02 | 3.59E-05 |
| *ASPG* | mRNA | 0.29 | 3.84E-02 | 4.74E-05 | | -0.44 | 1.33E-02 | 1.65E-05 |
| *ATP4B* | mRNA | 0.28 | 2.40E-03 | 2.96E-06 | | 1.16 | 2.34E-02 | 2.89E-05 |
| *B3GALT5* | mRNA | 0.27 | 7.22E-04 | 8.92E-07 | | 0.42 | 3.70E-03 | 9.14E-06 |
| *BCAN* | mRNA | 0.28 | 1.56E-02 | 1.93E-05 | | 0.43 | 1.55E-02 | 1.91E-05 |
| *BCL11A* | mRNA | -0.36 | 8.64E-03 | 1.07E-05 | | -0.30 | 6.43E-03 | 7.94E-06 |
| *BMP5* | mRNA | -0.39 | 2.96E-02 | 3.66E-05 | | 0.26 | 1.84E-02 | 4.54E-05 |
| *BNIPL* | mRNA | 0.41 | 6.72E-04 | 8.30E-07 | | -0.33 | 8.47E-03 | 1.05E-05 |
| *C12orf74* | mRNA | 0.28 | 4.11E-02 | 5.07E-05 | | -0.53 | 6.15E-03 | 1.52E-05 |
| *C8orf48* | mRNA | -0.64 | 4.87E-03 | 6.02E-06 | | 0.36 | 1.89E-02 | 2.33E-05 |
| *CALR3* | mRNA | -0.30 | 2.58E-03 | 3.18E-06 | | -0.42 | 2.11E-02 | 5.21E-05 |
| *CATSPER4* | mRNA | 0.36 | 3.51E-02 | 4.34E-05 | | -1.48 | 8.14E-03 | 1.00E-05 |
| *CCDC38* | mRNA | 0.27 | 2.03E-03 | 2.50E-06 | | -0.44 | 4.95E-03 | 1.22E-05 |
| *CD300LG* | mRNA | 0.34 | 1.51E-02 | 1.86E-05 | | 0.49 | 1.03E-02 | 2.54E-05 |
| *CECR2* | mRNA | 0.29 | 2.42E-02 | 2.99E-05 | | -0.30 | 6.15E-03 | 1.52E-05 |
| *CGB7* | mRNA | -0.34 | 5.19E-04 | 6.40E-07 | | -0.44 | 2.25E-02 | 2.78E-05 |
| *CIB4* | mRNA | 0.30 | 4.14E-02 | 5.11E-05 | | 0.50 | 2.93E-02 | 7.24E-05 |
| *CITED1* | mRNA | -0.80 | 1.70E-02 | 2.10E-05 | | 0.27 | 6.15E-03 | 1.52E-05 |
| *CNTN1* | mRNA | 0.61 | 1.76E-02 | 2.17E-05 | | -0.35 | 2.65E-03 | 6.53E-06 |
| *COL4A6* | mRNA | -0.40 | 1.80E-02 | 2.22E-05 | | -0.37 | 4.73E-02 | 5.84E-05 |
| *CPA2* | mRNA | 0.84 | 1.18E-02 | 1.46E-05 | | 0.27 | 2.80E-02 | 6.92E-05 |
| *CRB1* | mRNA | 0.31 | 1.71E-02 | 2.11E-05 | | 0.61 | 5.80E-03 | 1.43E-05 |
| *CSTL1* | mRNA | 0.30 | 9.05E-03 | 1.12E-05 | | 0.45 | 1.05E-02 | 2.58E-05 |
| *CTNNA2* | mRNA | -0.35 | 4.49E-02 | 5.54E-05 | | 0.79 | 5.80E-03 | 1.43E-05 |
| *CXCL5* | mRNA | -0.31 | 3.89E-02 | 4.80E-05 | | 0.32 | 6.80E-03 | 1.68E-05 |
| *CYP2S1* | mRNA | 0.42 | 3.97E-03 | 4.91E-06 | | -0.30 | 1.19E-02 | 1.47E-05 |
| *DGKA* | mRNA | 0.33 | 1.53E-02 | 1.89E-05 | | -0.27 | 1.95E-03 | 2.41E-06 |
| *DLX5* | mRNA | 0.38 | 9.99E-03 | 1.23E-05 | | -0.38 | 3.76E-03 | 4.65E-06 |
| *DNAH11* | mRNA | -0.35 | 1.64E-02 | 2.03E-05 | | 0.30 | 6.25E-03 | 1.55E-05 |
| *DNASE2B* | mRNA | 0.27 | 1.83E-03 | 2.27E-06 | | -0.50 | 6.85E-03 | 1.69E-05 |
| *ELAVL4* | mRNA | 0.28 | 8.13E-04 | 1.00E-06 | | 0.38 | 5.85E-03 | 1.44E-05 |
| *ELFN2* | mRNA | 0.28 | 4.43E-02 | 5.47E-05 | | 0.32 | 9.10E-03 | 2.25E-05 |
| *ELMOD1* | mRNA | 0.48 | 3.39E-02 | 4.18E-05 | | -0.38 | 6.90E-03 | 1.70E-05 |
| *ERAS* | mRNA | 0.27 | 4.61E-02 | 5.69E-05 | | -0.77 | 2.84E-03 | 7.01E-06 |
| *EVPLL* | mRNA | 0.27 | 6.91E-04 | 8.53E-07 | | -0.28 | 1.82E-02 | 4.48E-05 |
| *FAM129C* | mRNA | 0.27 | 4.49E-02 | 5.54E-05 | | -0.29 | 1.05E-02 | 2.58E-05 |
| *FAM181B* | mRNA | 0.47 | 1.74E-02 | 2.15E-05 | | -0.37 | 3.55E-03 | 8.76E-06 |
| *FAM83C* | mRNA | 0.30 | 6.82E-03 | 8.42E-06 | | -0.63 | 1.26E-02 | 1.56E-05 |
| *FBXO40* | mRNA | 0.30 | 2.49E-03 | 3.08E-06 | | -0.28 | 3.82E-02 | 9.41E-05 |
| *FGFBP1* | mRNA | 0.33 | 2.99E-02 | 3.69E-05 | | -0.28 | 5.05E-03 | 1.25E-05 |
| *FNDC7* | mRNA | 0.48 | 3.04E-03 | 3.75E-06 | | -1.03 | 4.41E-02 | 5.44E-05 |
| *GAD1* | mRNA | -0.30 | 3.24E-02 | 4.00E-05 | | -0.27 | 1.35E-02 | 3.33E-05 |
| *GDPD2* | mRNA | 0.28 | 1.64E-02 | 2.02E-05 | | -0.32 | 8.75E-03 | 2.16E-05 |
| *GHR* | mRNA | 0.85 | 1.91E-02 | 2.36E-05 | | -0.33 | 3.05E-03 | 7.53E-06 |
| *GHRHR* | mRNA | 0.30 | 1.12E-02 | 1.38E-05 | | 0.88 | 5.35E-03 | 1.33E-05 |
| *GJB1* | mRNA | 0.28 | 3.15E-02 | 3.89E-05 | | 0.53 | 3.18E-03 | 7.84E-06 |
| *GLIS1* | mRNA | 0.31 | 4.09E-03 | 5.05E-06 | | -0.29 | 7.55E-03 | 1.87E-05 |
| *GNGT1* | mRNA | 0.30 | 2.49E-03 | 3.07E-06 | | -0.28 | 1.25E-02 | 3.08E-05 |
| *GNRH2* | mRNA | 0.31 | 1.99E-02 | 2.45E-05 | | -0.48 | 3.98E-03 | 9.81E-06 |
| *GPR39* | mRNA | -0.33 | 4.44E-02 | 5.49E-05 | | 0.29 | 1.20E-02 | 2.96E-05 |
| *GPR62* | mRNA | 0.27 | 3.73E-02 | 4.61E-05 | | -0.39 | 1.22E-02 | 3.00E-05 |
| *HEPACAM2* | mRNA | 0.31 | 3.42E-04 | 4.22E-07 | | -0.29 | 3.23E-02 | 7.98E-05 |
| *HIST1H2BC* | mRNA | -0.35 | 2.68E-02 | 3.31E-05 | | 0.27 | 3.29E-02 | 4.07E-05 |
| *HIST1H3B* | mRNA | 0.76 | 1.23E-02 | 1.51E-05 | | -0.38 | 9.65E-03 | 2.38E-05 |
| *HIST1H3G* | mRNA | 0.82 | 2.04E-02 | 2.52E-05 | | -0.47 | 3.60E-02 | 4.44E-05 |
| *HIST1H4B* | mRNA | -0.27 | 4.72E-02 | 5.83E-05 | | 0.30 | 2.83E-02 | 6.99E-05 |
| *HIST1H4J* | mRNA | 0.38 | 3.16E-02 | 3.91E-05 | | -0.31 | 1.74E-02 | 4.28E-05 |
| *HORMAD2* | mRNA | 0.33 | 2.29E-02 | 2.83E-05 | | -0.42 | 3.27E-02 | 8.07E-05 |
| *HOXA13* | mRNA | 0.95 | 1.45E-02 | 1.79E-05 | | -0.47 | 4.49E-03 | 1.11E-05 |
| *HOXD13* | mRNA | 0.38 | 3.97E-02 | 4.90E-05 | | -0.31 | 2.53E-02 | 6.23E-05 |
| *HSD3B2* | mRNA | 0.28 | 1.42E-02 | 1.76E-05 | | -0.29 | 4.19E-02 | 1.03E-04 |
| *HSPB2* | mRNA | 0.35 | 2.24E-02 | 2.77E-05 | | -0.28 | 3.09E-02 | 3.81E-05 |
| *IGF2BP1* | mRNA | -0.36 | 2.47E-02 | 3.05E-05 | | -0.26 | 2.73E-02 | 6.74E-05 |
| *IYD* | mRNA | 0.34 | 2.73E-02 | 3.37E-05 | | -0.44 | 9.15E-03 | 2.25E-05 |
| *IZUMO1* | mRNA | 0.28 | 4.05E-02 | 5.00E-05 | | -0.42 | 2.02E-02 | 2.49E-05 |
| *JSRP1* | mRNA | 0.27 | 1.17E-02 | 1.45E-05 | | -0.26 | 7.25E-03 | 1.79E-05 |
| *KCNJ16* | mRNA | 0.30 | 1.84E-02 | 2.27E-05 | | -0.55 | 3.87E-03 | 9.55E-06 |
| *KLHL32* | mRNA | 0.29 | 1.94E-02 | 2.40E-05 | | 0.28 | 1.48E-02 | 3.64E-05 |
| *KLHL4* | mRNA | 0.49 | 2.91E-02 | 3.59E-05 | | 0.27 | 1.88E-02 | 4.64E-05 |
| *KRT32* | mRNA | 0.27 | 4.15E-02 | 5.12E-05 | | -0.72 | 1.97E-02 | 2.43E-05 |
| *KRT33A* | mRNA | 0.34 | 1.68E-04 | 2.08E-07 | | -0.98 | 3.13E-03 | 3.86E-06 |
| *KRT6A* | mRNA | 0.29 | 4.40E-02 | 5.43E-05 | | -0.34 | 2.71E-02 | 3.34E-05 |
| *L1CAM* | mRNA | 0.32 | 4.65E-03 | 5.74E-06 | | 0.37 | 3.85E-02 | 4.75E-05 |
| *LCN6* | mRNA | 0.28 | 1.54E-02 | 1.90E-05 | | -0.43 | 2.71E-02 | 6.70E-05 |
| *LGSN* | mRNA | 0.28 | 2.86E-02 | 3.53E-05 | | 0.72 | 1.96E-02 | 2.41E-05 |
| *LIMCH1* | mRNA | -0.39 | 3.87E-03 | 4.78E-06 | | 0.27 | 3.72E-02 | 4.60E-05 |
| *LINGO2* | mRNA | 0.26 | 3.94E-03 | 4.87E-06 | | 0.56 | 3.66E-03 | 9.02E-06 |
| *LIPC* | mRNA | 0.29 | 3.09E-03 | 3.81E-06 | | 0.29 | 6.40E-03 | 1.58E-05 |
| *LRAT* | mRNA | -0.52 | 4.69E-03 | 5.79E-06 | | 0.27 | 1.70E-02 | 4.20E-05 |
| *LRRC20* | mRNA | -0.27 | 1.40E-03 | 1.73E-06 | | 0.28 | 2.70E-05 | 3.33E-08 |
| *LTF* | mRNA | 0.49 | 2.78E-02 | 3.43E-05 | | -0.27 | 1.70E-02 | 4.19E-05 |
| *LUC7L2* | mRNA | -0.30 | 1.03E-03 | 1.27E-06 | | -0.48 | 2.71E-02 | 6.68E-05 |
| *MMP20* | mRNA | 0.27 | 7.47E-03 | 9.22E-06 | | -0.66 | 9.05E-03 | 2.23E-05 |
| *MMP27* | mRNA | 0.31 | 9.34E-03 | 1.15E-05 | | -1.37 | 7.74E-03 | 9.56E-06 |
| *MYO3A* | mRNA | 0.39 | 5.74E-03 | 7.09E-06 | | 0.45 | 8.65E-03 | 2.14E-05 |
| *NKX6-3* | mRNA | 0.27 | 2.69E-02 | 3.32E-05 | | -1.15 | 3.61E-02 | 4.46E-05 |
| *NLRP11* | mRNA | 0.55 | 1.40E-02 | 1.73E-05 | | -0.41 | 2.21E-02 | 5.45E-05 |
| *NPHP3* | mRNA | 0.28 | 4.97E-02 | 6.14E-05 | | -0.28 | 1.43E-03 | 1.76E-06 |
| *NUP210L* | mRNA | 0.27 | 7.12E-03 | 8.79E-06 | | -0.32 | 1.56E-02 | 3.84E-05 |
| *NXPH2* | mRNA | 0.30 | 4.02E-02 | 4.96E-05 | | 0.52 | 2.46E-02 | 6.08E-05 |
| *OBSCN* | mRNA | 0.27 | 1.79E-02 | 2.21E-05 | | -0.32 | 9.51E-04 | 1.17E-06 |
| *OR2B6* | mRNA | 0.31 | 3.02E-03 | 3.73E-06 | | 0.33 | 1.72E-02 | 4.25E-05 |
| *OR51E2* | mRNA | 0.29 | 1.62E-02 | 2.00E-05 | | 0.32 | 3.21E-02 | 7.92E-05 |
| *PABPC1L* | mRNA | -0.27 | 2.16E-02 | 2.67E-05 | | -0.28 | 2.33E-02 | 2.88E-05 |
| *PF4V1* | mRNA | 0.27 | 2.53E-02 | 3.13E-05 | | -0.84 | 2.79E-03 | 6.89E-06 |
| *PKP1* | mRNA | 0.30 | 4.34E-02 | 5.36E-05 | | -0.40 | 3.75E-03 | 4.63E-06 |
| *PLD1* | mRNA | -0.35 | 4.63E-02 | 5.72E-05 | | -0.29 | 2.78E-03 | 3.43E-06 |
| *PLEKHB1* | mRNA | 0.27 | 2.99E-03 | 3.69E-06 | | 0.33 | 7.73E-03 | 9.55E-06 |
| *PNMA2* | mRNA | 0.37 | 2.32E-02 | 2.87E-05 | | 0.27 | 4.89E-03 | 1.21E-05 |
| *POU2AF1* | mRNA | 0.38 | 2.04E-03 | 2.52E-06 | | -0.28 | 3.84E-03 | 9.47E-06 |
| *PRICKLE4* | mRNA | -0.29 | 4.45E-03 | 5.50E-06 | | -0.33 | 3.07E-03 | 7.57E-06 |
| *PRKAG3* | mRNA | 0.28 | 5.48E-03 | 6.76E-06 | | 0.36 | 2.61E-02 | 6.43E-05 |
| *PRND* | mRNA | 0.28 | 3.41E-03 | 4.21E-06 | | -0.30 | 2.45E-02 | 6.04E-05 |
| *PTHLH* | mRNA | -0.40 | 1.96E-03 | 2.42E-06 | | -0.27 | 1.18E-02 | 2.91E-05 |
| *RETN* | mRNA | 0.27 | 2.04E-02 | 2.52E-05 | | 0.32 | 1.38E-02 | 3.41E-05 |
| *RNF207* | mRNA | 0.51 | 3.80E-02 | 4.69E-05 | | -0.32 | 2.99E-03 | 3.70E-06 |
| *RTP1* | mRNA | 0.42 | 9.65E-03 | 1.19E-05 | | -0.44 | 2.35E-02 | 5.79E-05 |
| *RTP3* | mRNA | 0.29 | 4.02E-02 | 4.97E-05 | | -0.71 | 1.36E-02 | 3.35E-05 |
| *SERPINA3* | mRNA | 0.32 | 3.17E-02 | 3.92E-05 | | 0.46 | 1.52E-02 | 3.74E-05 |
| *SGCG* | mRNA | 0.38 | 5.19E-03 | 6.41E-06 | | -0.28 | 2.25E-02 | 5.56E-05 |
| *SGCZ* | mRNA | 0.27 | 4.62E-02 | 5.70E-05 | | 0.42 | 3.17E-02 | 7.81E-05 |
| *SLC17A3* | mRNA | 0.33 | 2.58E-02 | 3.19E-05 | | -0.52 | 2.70E-02 | 6.65E-05 |
| *SLC17A8* | mRNA | -0.33 | 4.36E-02 | 5.38E-05 | | 0.64 | 1.22E-02 | 3.00E-05 |
| *SLC2A14* | mRNA | 0.28 | 3.54E-03 | 4.37E-06 | | 0.33 | 1.18E-02 | 2.90E-05 |
| *SORBS2* | mRNA | 0.32 | 3.47E-02 | 4.29E-05 | | 0.32 | 4.65E-02 | 5.74E-05 |
| *SP5* | mRNA | -0.46 | 9.41E-03 | 1.16E-05 | | 0.35 | 1.33E-02 | 3.27E-05 |
| *ST6GALNAC1* | mRNA | 0.42 | 1.63E-02 | 2.02E-05 | | -0.33 | 2.05E-02 | 2.53E-05 |
| *TACSTD2* | mRNA | 0.77 | 7.60E-04 | 9.38E-07 | | -0.27 | 1.59E-02 | 1.96E-05 |
| *TCF15* | mRNA | 0.58 | 3.01E-02 | 3.71E-05 | | -0.35 | 4.79E-03 | 1.18E-05 |
| *THNSL2* | mRNA | 0.38 | 5.15E-04 | 6.36E-07 | | -0.30 | 3.59E-03 | 8.86E-06 |
| *TMEFF1* | mRNA | 0.61 | 3.77E-02 | 4.65E-05 | | 0.39 | 1.52E-02 | 3.75E-05 |
| *TMEM74* | mRNA | 0.30 | 6.86E-03 | 8.47E-06 | | 0.37 | 6.40E-03 | 1.58E-05 |
| *TNFRSF17* | mRNA | 0.30 | 2.37E-02 | 2.93E-05 | | -0.28 | 1.34E-02 | 3.30E-05 |
| *TRDN* | mRNA | 0.28 | 2.17E-02 | 2.68E-05 | | 0.29 | 3.25E-02 | 8.02E-05 |
| *TRIM7* | mRNA | 0.30 | 3.10E-02 | 3.83E-05 | | -0.32 | 3.36E-03 | 8.29E-06 |
| *TRIML1* | mRNA | 0.32 | 2.09E-02 | 2.58E-05 | | 0.75 | 1.55E-02 | 3.83E-05 |
| *TRPM5* | mRNA | 0.49 | 2.20E-02 | 2.72E-05 | | -0.28 | 2.58E-02 | 6.36E-05 |
| *TSNAXIP1* | mRNA | -0.39 | 9.57E-03 | 1.18E-05 | | -0.29 | 3.48E-03 | 8.58E-06 |
| *TSPAN19* | mRNA | -0.43 | 3.11E-02 | 3.84E-05 | | -0.33 | 3.18E-02 | 7.85E-05 |
| *TULP1* | mRNA | 0.27 | 2.19E-02 | 2.70E-05 | | -0.43 | 5.16E-03 | 6.37E-06 |
| *UGT2B28* | mRNA | 0.26 | 1.56E-02 | 1.93E-05 | | -1.65 | 5.50E-03 | 6.79E-06 |
| *VGF* | mRNA | -0.28 | 1.02E-02 | 1.26E-05 | | 0.46 | 6.34E-03 | 7.83E-06 |
| *WFDC10B* | mRNA | 0.31 | 9.87E-03 | 1.22E-05 | | -0.31 | 2.32E-02 | 5.73E-05 |
| *WFDC5* | mRNA | 0.34 | 4.30E-02 | 5.31E-05 | | -0.51 | 3.08E-02 | 3.80E-05 |
| *WNT4* | mRNA | 0.27 | 3.76E-02 | 4.64E-05 | | -0.30 | 4.20E-02 | 5.18E-05 |
| *ZMAT4* | mRNA | 0.54 | 3.29E-02 | 4.07E-05 | | 0.41 | 2.17E-02 | 5.34E-05 |
| *ZNF596* | mRNA | -0.40 | 2.26E-03 | 2.79E-06 | | -0.29 | 6.77E-03 | 8.36E-06 |
| *ZNF878* | mRNA | -0.75 | 2.18E-02 | 2.69E-05 | | 0.27 | 2.27E-02 | 5.61E-05 |

FC, fold change. FDR, false discovery rate.
