# Supplementary material for: A prognostic risk model based on DNA methylation levels of genes and lncRNAs in lung squamous cell carcinoma
Source: PeerJ. 2022 Mar 24;10:e13057. doi: 10.7717/peerj.13057 (PMC8958968; doi:10.7717/peerj.13057)
Supplement: Supplemental Information 2 [file peerj-10-13057-s002.docx]

| Table S2. The methylation level of the 18 genes in the GSE39279 dataset. | | | | | | |  |
| --- | --- | --- | --- | --- | --- | --- | --- |
| **Symbol** | **Type** | **Locus** | **Chr** | **Position** | **logFC** | **FDR** | **Pvalue** |
| RMST | lncRNA | cg27315341 | chr12 | 96411165 | 0.65 | 5.49E-03 | 2.17E-05 |
| DIRC3 | lncRNA | cg26967619 | chr2 | 218329742 | 0.57 | 7.68E-03 | 3.16E-03 |
| LIMCH1 | mRNA | cg26248284 | chr4 | 41057610 | -0.78 | 4.29E-03 | 3.13E-05 |
| NPHP3 | mRNA | cg21574574 | chr3 | 133883336 | 0.91 | 4.28E-02 | 6.41E-05 |
| ADH7 | mRNA | cg14095316 | chr4 | 100575561 | 0.58 | 2.53E-03 | 3.27E-04 |
| TRIM7 | mRNA | cg26677546 | chr5 | 180563040 | 0.34 | 3.13E-03 | 3.24E-05 |
| GNRH2 | mRNA | cg23848712 | chr20 | 2970992 | 0.79 | 2.13E-02 | 2.44E-05 |
| WFDC10B | mRNA | cg22988566 | chr20 | 43768283 | 0.62 | 1.94E-03 | 1.38E-04 |
| DGKA | mRNA | cg26477856 | chr12 | 54611182 | 0.63 | 1.73E-03 | 3.41E-03 |
| HORMAD2 | mRNA | cg24211826 | chr22 | 28902326 | 0.62 | 2.13E-04 | 1.04E-03 |
| ABCA12 | mRNA | cg26843807 | chr2 | 215595615 | 0.48 | 3.26E-02 | 3.43E-03 |
| SGCG | mRNA | cg13168042 | chr13 | 22653059 | 0.36 | 2.37E-03 | 3.47E-04 |
| THNSL2 | mRNA | cg24977027 | chr2 | 88250462 | 0.48 | 1.05E-03 | 3.46E-05 |
| BNIPL | mRNA | cg15380890 | chr1 | 149275595 | 0.47 | 3.67E-03 | 3.76E-05 |
| ST6GALNAC1 | mRNA | cg26550194 | chr17 | 72151523 | 0.32 | 2.65E-02 | 2.08E-04 |
| RTP1 | mRNA | cg16759976 | chr3 | 188397414 | 0.64 | 1.93E-03 | 2.72E-03 |
| FAM181B | mRNA | cg24532471 | chr11 | 82122720 | 0.67 | 1.24E-03 | 6.77E-04 |
| LTF | mRNA | cg27314002 | chr3 | 46481523 | 0.74 | 2.14E-03 | 5.04E-03 |
