# Supplementary material for: A prognostic risk model based on DNA methylation levels of genes and lncRNAs in lung squamous cell carcinoma
Source: PeerJ. 2022 Mar 24;10:e13057. doi: 10.7717/peerj.13057 (PMC8958968; doi:10.7717/peerj.13057)
Supplement: Supplemental Information 3 [file peerj-10-13057-s003.docx]

Table S3. List of the 41 genes with differentially expression and methylation levels in three weighted correlation network analysis co-methylation modules.

| **Methylation level** | | | | | **Expression level** | | |
| --- | --- | --- | --- | --- | --- | --- | --- |
| **Symbol** | **Color** | **logFC** | **FDR** | **P value** | **logFC** | **FDR** | **P value** |
| *ABCA12* | turquoise | 0.35 | 2.36E-02 | 2.92E-05 | -0.29 | 5.20E-03 | 1.29E-05 |
| *ADH7* | turquoise | 0.30 | 3.81E-02 | 4.71E-05 | -0.57 | 2.18E-02 | 2.70E-05 |
| *AMPD1* | pink | 0.29 | 1.53E-02 | 1.89E-05 | -0.48 | 4.55E-03 | 1.12E-05 |
| *ASPG* | turquoise | 0.29 | 3.84E-02 | 4.74E-05 | -0.44 | 1.33E-02 | 1.65E-05 |
| *BNIPL* | turquoise | 0.41 | 6.72E-04 | 8.30E-07 | -0.33 | 8.47E-03 | 1.05E-05 |
| *CECR2* | turquoise | 0.29 | 2.42E-02 | 2.99E-05 | -0.30 | 6.15E-03 | 1.52E-05 |
| *CIB4* | turquoise | 0.30 | 4.14E-02 | 5.11E-05 | 0.50 | 2.93E-02 | 7.24E-05 |
| *CRB1* | turquoise | 0.31 | 1.71E-02 | 2.11E-05 | 0.61 | 5.80E-03 | 1.43E-05 |
| *DGKA* | turquoise | 0.33 | 1.53E-02 | 1.89E-05 | -0.27 | 1.95E-03 | 2.41E-06 |
| *ELFN2* | pink | 0.28 | 4.43E-02 | 5.47E-05 | 0.32 | 9.10E-03 | 2.25E-05 |
| *FAM181B* | turquoise | 0.47 | 1.74E-02 | 2.15E-05 | -0.37 | 3.55E-03 | 8.76E-06 |
| *FNDC7* | turquoise | 0.48 | 3.04E-03 | 3.75E-06 | -1.03 | 4.41E-02 | 5.44E-05 |
| *GNRH2* | turquoise | 0.31 | 1.99E-02 | 2.45E-05 | -0.48 | 3.98E-03 | 9.81E-06 |
| *HORMAD2* | turquoise | 0.33 | 2.29E-02 | 2.83E-05 | -0.42 | 3.27E-02 | 8.07E-05 |
| *HSPB2* | turquoise | 0.35 | 2.24E-02 | 2.77E-05 | -0.28 | 3.09E-02 | 3.81E-05 |
| *IYD* | turquoise | 0.34 | 2.73E-02 | 3.37E-05 | -0.44 | 9.15E-03 | 2.25E-05 |
| *KCNJ16* | turquoise | 0.30 | 1.84E-02 | 2.27E-05 | -0.55 | 3.87E-03 | 9.55E-06 |
| *KLHL32* | pink | 0.29 | 1.94E-02 | 2.40E-05 | 0.28 | 1.48E-02 | 3.64E-05 |
| *KRT6A* | turquoise | 0.29 | 4.40E-02 | 5.43E-05 | -0.34 | 2.71E-02 | 3.34E-05 |
| *L1CAM* | pink | 0.32 | 4.65E-03 | 5.74E-06 | 0.37 | 3.85E-02 | 4.75E-05 |
| *LIMCH1* | turquoise | -0.39 | 3.87E-03 | 4.78E-06 | 0.27 | 3.72E-02 | 4.60E-05 |
| *LRAT* | green | -0.52 | 4.69E-03 | 5.79E-06 | 0.27 | 1.70E-02 | 4.20E-05 |
| *LTF* | pink | 0.49 | 2.78E-02 | 3.43E-05 | -0.27 | 1.70E-02 | 4.19E-05 |
| *MYO3A* | pink | 0.39 | 5.74E-03 | 7.09E-06 | 0.45 | 8.65E-03 | 2.14E-05 |
| *NPHP3* | turquoise | 0.28 | 4.97E-02 | 6.14E-05 | -0.28 | 1.43E-03 | 1.76E-06 |
| *NXPH2* | pink | 0.30 | 4.02E-02 | 4.96E-05 | 0.52 | 2.46E-02 | 6.08E-05 |
| *PLD1* | turquoise | -0.35 | 4.63E-02 | 5.72E-05 | -0.29 | 2.78E-03 | 3.43E-06 |
| *PTHLH* | green | -0.40 | 1.96E-03 | 2.42E-06 | -0.27 | 1.18E-02 | 2.91E-05 |
| *RTP1* | turquoise | 0.42 | 9.65E-03 | 1.19E-05 | -0.44 | 2.35E-02 | 5.79E-05 |
| *SGCG* | pink | 0.38 | 5.19E-03 | 6.41E-06 | -0.28 | 2.25E-02 | 5.56E-05 |
| *SORBS2* | turquoise | 0.32 | 3.47E-02 | 4.29E-05 | 0.32 | 4.65E-02 | 5.74E-05 |
| *SP5* | turquoise | -0.46 | 9.41E-03 | 1.16E-05 | 0.35 | 1.33E-02 | 3.27E-05 |
| *ST6GALNAC1* | turquoise | 0.42 | 1.63E-02 | 2.02E-05 | -0.33 | 2.05E-02 | 2.53E-05 |
| *THNSL2* | turquoise | 0.38 | 5.15E-04 | 6.36E-07 | -0.30 | 3.59E-03 | 8.86E-06 |
| *TNFRSF17* | pink | 0.30 | 2.37E-02 | 2.93E-05 | -0.28 | 1.34E-02 | 3.30E-05 |
| *TRIM7* | turquoise | 0.30 | 3.10E-02 | 3.83E-05 | -0.32 | 3.36E-03 | 8.29E-06 |
| *TRIML1* | pink | 0.32 | 2.09E-02 | 2.58E-05 | 0.75 | 1.55E-02 | 3.83E-05 |
| *TSNAXIP1* | green | -0.39 | 9.57E-03 | 1.18E-05 | -0.29 | 3.48E-03 | 8.58E-06 |
| *WFDC10B* | turquoise | 0.31 | 9.87E-03 | 1.22E-05 | -0.31 | 2.32E-02 | 5.73E-05 |
| *WFDC5* | turquoise | 0.34 | 4.30E-02 | 5.31E-05 | -0.51 | 3.08E-02 | 3.80E-05 |
| *ZNF596* | green | -0.40 | 2.26E-03 | 2.79E-06 | -0.29 | 6.77E-03 | 8.36E-06 |

FC, fold change. FDR, false discovery rate.
