# Supplementary material for: A prognostic risk model based on DNA methylation levels of genes and lncRNAs in lung squamous cell carcinoma
Source: PeerJ. 2022 Mar 24;10:e13057. doi: 10.7717/peerj.13057 (PMC8958968; doi:10.7717/peerj.13057)
Supplement: Supplemental Information 4 [file peerj-10-13057-s004.docx]

Table S4. RNAs associated with prognosis in lung squamous cell carcinoma by univariate Cox regression analysis and methylation level.

| **Symbol** | **type** | **β** | **Exp(β)** | **p** |
| --- | --- | --- | --- | --- |
| *DIO3OS* | lncRNA | 1.69 | 5.42 | 1.75E-02 |
| *RMST* | lncRNA | 1.06 | 2.89 | 2.95E-02 |
| *TCL6* | lncRNA | 1.56 | 4.75 | 3.66E-02 |
| *DIRC3* | lncRNA | 1.41 | 4.08 | 4.24E-02 |
| *BNIPL* | mRNA | 2.37 | 10.70 | 1.60E-04 |
| *ST6GALNAC1* | mRNA | 2.96 | 19.30 | 2.90E-04 |
| *RTP1* | mRNA | 1.62 | 5.06 | 9.80E-04 |
| *DGKA* | mRNA | 2.54 | 12.70 | 3.00E-03 |
| *LTF* | mRNA | 1.77 | 5.89 | 4.20E-03 |
| *FNDC7* | mRNA | 2.23 | 9.28 | 7.33E-03 |
| *HORMAD2* | mRNA | 1.42 | 4.14 | 7.40E-03 |
| *THNSL2* | mRNA | 1.39 | 4.02 | 8.70E-03 |
| *KRT6A* | mRNA | 2.01 | 7.50 | 9.00E-03 |
| *LIMCH1* | mRNA | -4.83 | 7.96E-03 | 1.07E-02 |
| *FAM181B* | mRNA | 2.95 | 19.20 | 1.44E-02 |
| *IYD* | mRNA | 1.41 | 4.12 | 1.50E-02 |
| *TRIM7* | mRNA | 1.48 | 4.38 | 1.80E-02 |
| *GNRH2* | mRNA | 1.46 | 4.31 | 2.30E-02 |
| *WFDC10B* | mRNA | 1.69 | 5.42 | 2.43E-02 |
| *SGCG* | mRNA | 1.14 | 3.12 | 2.94E-02 |
| *NPHP3* | mRNA | 1.30 | 3.69 | 2.91E-02 |
| *ADH7* | mRNA | 1.34 | 3.84 | 3.00E-02 |
| *CECR2* | mRNA | 1.22 | 3.40 | 3.08E-02 |
| *ABCA12* | mRNA | 1.34 | 3.80 | 3.21E-02 |
| *TNFRSF17* | mRNA | 1.45 | 4.24 | 3.40E-02 |
| *WFDC5* | mRNA | 3.50 | 33.10 | 4.12E-02 |
