# Supplementary material for: A prognostic risk model based on DNA methylation levels of genes and lncRNAs in lung squamous cell carcinoma
Source: PeerJ. 2022 Mar 24;10:e13057. doi: 10.7717/peerj.13057 (PMC8958968; doi:10.7717/peerj.13057)
Supplement: Supplemental Information 5 [file peerj-10-13057-s005.docx]

Table S5. The lncRNA-mRNA pairs for DIRC3 and RMST with confident correlations (Pearson correlation coefficient, r >0.4 or < -0.4).

| lncRNA | mRNA | r |
| --- | --- | --- |
| *DIRC3* | *DUSP4* | -0.53 |
| *DIRC3* | *BSPRY* | -0.52 |
| *DIRC3* | *PLEKHB1* | -0.48 |
| *RMST* | *S1PR5* | -0.48 |
| *DIRC3* | *GPR39* | -0.47 |
| *DIRC3* | *LPCAT1* | -0.46 |
| *DIRC3* | *SORBS2* | -0.46 |
| *DIRC3* | *DOK7* | -0.46 |
| *DIRC3* | *CAMK2N1* | -0.45 |
| *RMST* | *TMEM40* | -0.45 |
| *DIRC3* | *NFE2* | -0.45 |
| *DIRC3* | *HTR1D* | -0.45 |
| *RMST* | *KRT6B* | -0.44 |
| *RMST* | *FGFBP1* | -0.44 |
| *RMST* | *TP63* | -0.44 |
| *RMST* | *KRT6A* | -0.44 |
| *RMST* | *PLD1* | -0.43 |
| *DIRC3* | *ALDH7A1* | -0.43 |
| *RMST* | *GJB5* | -0.43 |
| *RMST* | *ST6GALNAC2* | -0.43 |
| *DIRC3* | *EFHD1* | -0.42 |
| *DIRC3* | *QPCT* | -0.42 |
| *DIRC3* | *ZNF350* | -0.42 |
| *DIRC3* | *NT5E* | -0.42 |
| *RMST* | *FXYD3* | -0.42 |
| *RMST* | *KRT16* | -0.42 |
| *DIRC3* | *FCN2* | -0.42 |
| *RMST* | *GCLC* | -0.42 |
| *RMST* | *B3GNT4* | -0.41 |
| *RMST* | *GPR87* | -0.41 |
| *RMST* | *EPN3* | -0.41 |
| *DIRC3* | *ZNF613* | -0.41 |
| *RMST* | *DSG3* | -0.41 |
| *RMST* | *SRRM3* | -0.41 |
| *RMST* | *TMEM117* | -0.40 |
| *RMST* | *FOXE1* | -0.40 |
| *DIRC3* | *AIM2* | -0.40 |
| *RMST* | *LAT* | 0.40 |
| *DIRC3* | *STOX2* | 0.40 |
| *RMST* | *VWCE* | 0.40 |
| *DIRC3* | *FAM153A* | 0.40 |
| *DIRC3* | *ZNF750* | 0.40 |
| *DIRC3* | *FXYD3* | 0.40 |
| *RMST* | *DNASE2B* | 0.40 |
| *RMST* | *ELF5* | 0.40 |
| *DIRC3* | *DUOX1* | 0.40 |
| *DIRC3* | *SDR9C7* | 0.40 |
| *DIRC3* | *RTP3* | 0.40 |
| *DIRC3* | *CRYAB* | 0.40 |
| *DIRC3* | *LRP5L* | 0.40 |
| *RMST* | *ZCCHC12* | 0.41 |
| *RMST* | *MYH6* | 0.41 |
| *DIRC3* | *GABBR1* | 0.41 |
| *DIRC3* | *RELL2* | 0.41 |
| *DIRC3* | *WDR72* | 0.41 |
| *DIRC3* | *GDPD2* | 0.41 |
| *DIRC3* | *GHRHR* | 0.41 |
| *DIRC3* | *POF1B* | 0.41 |
| *RMST* | *GLB1L3* | 0.41 |
| *RMST* | *SRPK3* | 0.41 |
| *DIRC3* | *TNS4* | 0.41 |
| *RMST* | *ANKS1B* | 0.41 |
| *RMST* | *QPCT* | 0.41 |
| *RMST* | *ELMOD1* | 0.41 |
| *RMST* | *TRPV6* | 0.41 |
| *RMST* | *P2RX3* | 0.41 |
| *RMST* | *PPM1E* | 0.41 |
| *RMST* | *SPATA1* | 0.41 |
| *DIRC3* | *ACPP* | 0.42 |
| *RMST* | *NEIL1* | 0.42 |
| *DIRC3* | *KRT9* | 0.42 |
| *RMST* | *GRID2IP* | 0.42 |
| *RMST* | *FCN2* | 0.42 |
| *DIRC3* | *ITGB8* | 0.42 |
| *RMST* | *FNDC7* | 0.42 |
| *DIRC3* | *SNX31* | 0.42 |
| *RMST* | *TECTA* | 0.42 |
| *DIRC3* | *TMEM40* | 0.42 |
| *RMST* | *SNX32* | 0.42 |
| *RMST* | *CCDC153* | 0.42 |
| *DIRC3* | *GJB2* | 0.42 |
| *DIRC3* | *AIRE* | 0.42 |
| *DIRC3* | *PAX3* | 0.43 |
| *RMST* | *HPX* | 0.43 |
| *RMST* | *RASL10A* | 0.43 |
| *DIRC3* | *PTH2R* | 0.43 |
| *DIRC3* | *KRT82* | 0.43 |
| *DIRC3* | *TMPRSS4* | 0.43 |
| *RMST* | *NLRP11* | 0.43 |
| *RMST* | *ROBO3* | 0.43 |
| *RMST* | *ZNF578* | 0.43 |
| *RMST* | *RERG* | 0.43 |
| *DIRC3* | *HOXD13* | 0.43 |
| *DIRC3* | *NDUFA4L2* | 0.43 |
| *DIRC3* | *SERPINB13* | 0.44 |
| *DIRC3* | *GOLGA8A* | 0.44 |
| *RMST* | *EML5* | 0.44 |
| *DIRC3* | *CTNNAL1* | 0.44 |
| *DIRC3* | *KRT14* | 0.44 |
| *DIRC3* | *RAPGEFL1* | 0.44 |
| *DIRC3* | *DQX1* | 0.44 |
| *RMST* | *IGDCC3* | 0.44 |
| *RMST* | *POU5F1* | 0.44 |
| *RMST* | *MMEL1* | 0.44 |
| *RMST* | *C12orf74* | 0.44 |
| *RMST* | *TTLL3* | 0.44 |
| *DIRC3* | *CSTA* | 0.44 |
| *RMST* | *SLC25A34* | 0.44 |
| *DIRC3* | *CNTN1* | 0.45 |
| *DIRC3* | *RYR1* | 0.45 |
| *RMST* | *DNAH11* | 0.45 |
| *DIRC3* | *SLC22A9* | 0.45 |
| *DIRC3* | *ZNF711* | 0.45 |
| *RMST* | *ZNF613* | 0.45 |
| *DIRC3* | *PITX1* | 0.45 |
| *DIRC3* | *CPA2* | 0.45 |
| *RMST* | *FAM129C* | 0.45 |
| *DIRC3* | *SLC26A10* | 0.45 |
| *RMST* | *ATP4B* | 0.45 |
| *RMST* | *ARMC4* | 0.45 |
| *RMST* | *METTL7A* | 0.45 |
| *RMST* | *IYD* | 0.45 |
| *DIRC3* | *PMP2* | 0.45 |
| *DIRC3* | *RNF175* | 0.45 |
| *RMST* | *LCN6* | 0.45 |
| *DIRC3* | *FGFBP1* | 0.46 |
| *DIRC3* | *KRT6C* | 0.46 |
| *DIRC3* | *KRT16* | 0.46 |
| *DIRC3* | *CARD17* | 0.46 |
| *DIRC3* | *ATP13A5* | 0.46 |
| *DIRC3* | *KRTAP19.1* | 0.46 |
| *RMST* | *DPP4* | 0.46 |
| *DIRC3* | *WNT7B* | 0.46 |
| *DIRC3* | *SPRR1B* | 0.46 |
| *RMST* | *IGFN1* | 0.46 |
| *DIRC3* | *TTLL10* | 0.46 |
| *DIRC3* | *ST6GALNAC2* | 0.46 |
| *DIRC3* | *IZUMO1* | 0.46 |
| *RMST* | *SLC8A1* | 0.46 |
| *DIRC3* | *TCF15* | 0.46 |
| *DIRC3* | *SYT14* | 0.46 |
| *DIRC3* | *DLX1* | 0.46 |
| *RMST* | *C8orf48* | 0.47 |
| *DIRC3* | *LTB4R2* | 0.47 |
| *DIRC3* | *SEMA6C* | 0.47 |
| *DIRC3* | *KRT6B* | 0.47 |
| *DIRC3* | *DENND2C* | 0.47 |
| *RMST* | *OVCH2* | 0.47 |
| *RMST* | *AMT* | 0.47 |
| *RMST* | *DCX* | 0.47 |
| *DIRC3* | *SERPINB11* | 0.47 |
| *DIRC3* | *DSC3* | 0.47 |
| *DIRC3* | *SPRR4* | 0.47 |
| *DIRC3* | *EVPLL* | 0.47 |
| *DIRC3* | *TRDN* | 0.47 |
| *DIRC3* | *EML6* | 0.47 |
| *DIRC3* | *KRT32* | 0.47 |
| *DIRC3* | *OTX2* | 0.47 |
| *RMST* | *MYOZ1* | 0.48 |
| *DIRC3* | *GABRE* | 0.48 |
| *RMST* | *LRRC39* | 0.48 |
| *DIRC3* | *MMP27* | 0.48 |
| *DIRC3* | *KRT6A* | 0.48 |
| *DIRC3* | *WFDC5* | 0.48 |
| *DIRC3* | *CLCA2* | 0.48 |
| *DIRC3* | *MAPK10* | 0.48 |
| *RMST* | *CCDC152* | 0.48 |
| *DIRC3* | *RAD51AP2* | 0.48 |
| *DIRC3* | *GHR* | 0.48 |
| *RMST* | *HSD3B2* | 0.48 |
| *DIRC3* | *TECTA* | 0.48 |
| *DIRC3* | *GNB3* | 0.48 |
| *DIRC3* | *OTX1* | 0.48 |
| *DIRC3* | *TRIM29* | 0.49 |
| *RMST* | *DYNC2H1* | 0.49 |
| *RMST* | *GPC5* | 0.49 |
| *DIRC3* | *NEUROD2* | 0.49 |
| *DIRC3* | *FAM83C* | 0.49 |
| *DIRC3* | *CALML3* | 0.49 |
| *RMST* | *PARD3B* | 0.49 |
| *RMST* | *KCNJ3* | 0.49 |
| *DIRC3* | *SLC5A7* | 0.49 |
| *DIRC3* | *SHOX2* | 0.49 |
| *DIRC3* | *ZNF556* | 0.49 |
| *DIRC3* | *PGLYRP3* | 0.49 |
| *RMST* | *SLC17A3* | 0.49 |
| *DIRC3* | *ADAD2* | 0.49 |
| *RMST* | *SP5* | 0.49 |
| *DIRC3* | *KRT33A* | 0.50 |
| *RMST* | *SLC5A7* | 0.50 |
| *DIRC3* | *KRT17* | 0.50 |
| *DIRC3* | *SPRR2A* | 0.50 |
| *DIRC3* | *SLC4A11* | 0.50 |
| *DIRC3* | *IGSF3* | 0.50 |
| *DIRC3* | *SYNGR1* | 0.50 |
| *DIRC3* | *NTS* | 0.50 |
| *DIRC3* | *S1PR5* | 0.50 |
| *RMST* | *TSPAN19* | 0.50 |
| *RMST* | *ELFN2* | 0.50 |
| *DIRC3* | *IGSF11* | 0.50 |
| *DIRC3* | *RAB40B* | 0.50 |
| *DIRC3* | *GRK7* | 0.50 |
| *DIRC3* | *FEZF1* | 0.50 |
| *DIRC3* | *PLD1* | 0.51 |
| *RMST* | *NFE2* | 0.51 |
| *DIRC3* | *HLF* | 0.51 |
| *RMST* | *GPR39* | 0.51 |
| *DIRC3* | *ADAMTS17* | 0.51 |
| *DIRC3* | *XG* | 0.51 |
| *DIRC3* | *SLC15A1* | 0.51 |
| *RMST* | *NUP210L* | 0.51 |
| *DIRC3* | *LGI3* | 0.51 |
| *DIRC3* | *BDH1* | 0.51 |
| *DIRC3* | *BMP7* | 0.52 |
| *DIRC3* | *S100A5* | 0.52 |
| *DIRC3* | *C17orf99* | 0.52 |
| *DIRC3* | *FAM84A* | 0.52 |
| *RMST* | *KLHL32* | 0.52 |
| *DIRC3* | *UCN2* | 0.52 |
| *DIRC3* | *ARTN* | 0.52 |
| *DIRC3* | *KRT31* | 0.52 |
| *DIRC3* | *BNC1* | 0.53 |
| *RMST* | *LPCAT1* | 0.53 |
| *RMST* | *TSNAXIP1* | 0.53 |
| *RMST* | *VWA2* | 0.53 |
| *DIRC3* | *GSTA1* | 0.53 |
| *DIRC3* | *SUSD4* | 0.53 |
| *DIRC3* | *DSG3* | 0.53 |
| *DIRC3* | *PNCK* | 0.53 |
| *RMST* | *FOXA2* | 0.53 |
| *DIRC3* | *BCL11A* | 0.53 |
| *DIRC3* | *CYP2S1* | 0.54 |
| *DIRC3* | *GJB6* | 0.54 |
| *DIRC3* | *USH1G* | 0.54 |
| *RMST* | *CD300LG* | 0.54 |
| *DIRC3* | *GDA* | 0.54 |
| *DIRC3* | *DLX6* | 0.54 |
| *DIRC3* | *ERC2* | 0.54 |
| *DIRC3* | *GJB5* | 0.55 |
| *DIRC3* | *SGK1* | 0.55 |
| *DIRC3* | *TSHZ2* | 0.55 |
| *DIRC3* | *TMPRSS11D* | 0.55 |
| *DIRC3* | *SOX21* | 0.55 |
| *DIRC3* | *VANGL2* | 0.55 |
| *RMST* | *KCNQ3* | 0.55 |
| *DIRC3* | *SLCO1A2* | 0.55 |
| *DIRC3* | *FAM71F1* | 0.55 |
| *DIRC3* | *SRRM3* | 0.55 |
| *DIRC3* | *GSTM2* | 0.56 |
| *DIRC3* | *KRT5* | 0.56 |
| *DIRC3* | *PCSK9* | 0.56 |
| *DIRC3* | *RASSF9* | 0.57 |
| *DIRC3* | *DAPL1* | 0.57 |
| *DIRC3* | *COL4A6* | 0.57 |
| *DIRC3* | *ABCA13* | 0.58 |
| *DIRC3* | *WNT2B* | 0.58 |
| *DIRC3* | *PTHLH* | 0.58 |
| *DIRC3* | *COL7A1* | 0.59 |
| *DIRC3* | *TRPV4* | 0.59 |
| *DIRC3* | *TMEM117* | 0.59 |
| *RMST* | *GJB1* | 0.60 |
| *RMST* | *PNMA2* | 0.60 |
| *DIRC3* | *KRT15* | 0.60 |
| *RMST* | *PLEKHB1* | 0.60 |
| *DIRC3* | *TMPRSS11F* | 0.60 |
| *RMST* | *SORBS2* | 0.60 |
| *RMST* | *SLC17A8* | 0.60 |
| *DIRC3* | *TMPRSS11A* | 0.61 |
| *DIRC3* | *TP63* | 0.61 |
| *DIRC3* | *IRX6* | 0.61 |
| *DIRC3* | *P2RY1* | 0.61 |
| *RMST* | *CPB2* | 0.62 |
| *DIRC3* | *MSGN1* | 0.62 |
| *DIRC3* | *KLHL13* | 0.63 |
| *DIRC3* | *FOXE1* | 0.63 |
| *RMST* | *DCDC2* | 0.63 |
| *DIRC3* | *DLX5* | 0.63 |
| *DIRC3* | *WNT5A* | 0.64 |
| *RMST* | *BMP5* | 0.64 |
| *DIRC3* | *RGMA* | 0.65 |
| *DIRC3* | *EFS* | 0.66 |
| *DIRC3* | *SESN3* | 0.67 |
| *RMST* | *VEPH1* | 0.67 |
| *DIRC3* | *CYB5R2* | 0.68 |
| *DIRC3* | *PKP1* | 0.68 |
| *DIRC3* | *ARHGAP24* | 0.68 |
| *DIRC3* | *NTRK2* | 0.72 |
| *DIRC3* | *FGFR2* | 0.78 |
| *RTP1* | *DIRC3* | 0.75 |
| *ADH7* | *DIRC3* | 0.73 |
| *LTF* | *RMST* | 0.71 |
| *DGKA* | *DIRC3* | 0.69 |
| *SGCG* | *RMST* | 0.66 |
| *LIMCH1* | *RMST* | -0.65 |
| *FAM181B* | *DIRC3* | 0.63 |
| *SGCG* | *DIRC3* | 0.61 |
| *WFDC10B* | *RMST* | 0.59 |
| *NPHP3* | *DIRC3* | 0.55 |
| *TRIM7* | *DIRC3* | 0.55 |
| *ABCA12* | *DIRC3* | 0.54 |
| *GNRH2* | *RMST* | 0.53 |
| *NPHP3* | *RMST* | 0.53 |
| *GNRH2* | *DIRC3* | 0.51 |
| *BNIPL* | *DIRC3* | 0.51 |
| *THNSL2* | *RMST* | 0.49 |
| *HORMAD2* | *RMST* | 0.49 |
| *BNIPL* | *RMST* | 0.45 |
| *ST6GALNAC1* | *RMST* | 0.44 |
| *HORMAD2* | *DIRC3* | 0.43 |
| *RTP1* | *RMST* | 0.40 |
| *THNSL2* | *DIRC3* | -0.40 |
| *LTF* | *DIRC3* | -0.43 |
| *ST6GALNAC1* | *DIRC3* | -0.45 |
| *WFDC10B* | *DIRC3* | -0.46 |
| *FAM181B* | *RMST* | -0.50 |
| *LIMCH1* | *DIRC3* | -0.51 |
| *DGKA* | *RMST* | -0.52 |
| *ABCA12* | *RMST* | -0.52 |
| *ADH7* | *RMST* | -0.57 |
| *TRIM7* | *RMST* | -0.67 |
